# Genome and Secondary Metabolites Analysis of *Fusarium oxysporum* BPF55 Associated with *Blaps rynchopetera* and Its Anti-MRSA Biofilm Potential

**DOI:** 10.3390/jof12040236

**Published:** 2026-03-25

**Authors:** Xiaolu Zhu, Haorong Yin, Dasong Yang, Yinhe Yang

**Affiliations:** 1 Yunnan Provincial Key Laboratory of Entomological Biopharmaceutical R&D, Dali University, Dali 671000, China; 18288829369@163.com (X.Z.); yhr6208@163.com (H.Y.); 2 National-Local Joint Engineering Research Center of Entomoceutics, Dali University, Dali 671000, China; 3 College of Pharmacy, Dali University, Dali 671000, China

**Keywords:** whole genome sequencing, biosynthetic gene clusters, UPLC-MS/MS, *Fusarium oxysporum*, secondary metabolites, biofilm inhibition, molecular docking

## Abstract

Antimicrobial resistance (AMR) represents a critical global health challenge, with methicillin-resistant *Staphylococcus aureus* (MRSA) posing a significant threat in both hospital-acquired and community-associated infections. Research has demonstrated that biofilm formation is a key factor contributing to drug resistance in MRSA. In this study, we investigated an fungus, *Fusarium oxysporum* BPF55, isolated from *Blaps rynchopetera*, which inhibits MRSA biofilm formation. The aim of this research was to identify the fungal strain and comprehensively characterize its genomic features, as well as to evaluate its anti-MRSA biofilm potential. Whole-genome sequencing revealed a genome size of 50,097,681 base pairs, a GC content of 47.36%, and 16,507 predicted coding genes. AntiSMASH analysis identified 56 secondary metabolite biosynthetic gene clusters, including those involved in the synthesis of various natural products such as terpenes, non-ribosomal peptides, and polyketides. Using UPLC-MS/MS, 15 compounds were annotated from the ethyl acetate extract. Molecular docking studies demonstrated that four compounds exhibit varying affinities for SarA and AgrA, two key proteins involved in MRSA biofilm formation. Overall, these findings suggest that the fungus *F. oxysporum* BPA55 produces a variety of secondary metabolites and contains bioactive compounds with potential anti-MRSA biofilm activity.

## 1. Introduction

Antimicrobial resistance (AMR) refers to the increased resistance of bacteria to antibiotics, resulting in the loss of the drugs’ therapeutic effect on infections [1]. In less than 30 years, AMR could become deadlier than cancer, with an estimated 10 million people expected to die annually by 2050 [2]. Methicillin-resistant *Staphylococcus aureus* (MRSA) exhibits a broad spectrum of drug resistance, which not only makes treatment challenging but also increases the risk of potential complications. The formation of biofilms by MRSA prolongs clinical infections and enhances antibiotic resistance. Identifying natural inhibitors with novel structures and unique mechanisms of action is urgently needed to address this issue. Biofilms confer antimicrobial resistance through multiple mechanisms, including limited antibiotic penetration, genetic adaptation, altered growth rates and metabolism, antimicrobial neutralization, formation of persistent cells, and increased rates of gene transfer. Consequently, inhibiting biofilm formation has recently been recognized as a crucial therapeutic strategy for combating persistent bacterial infections [3].

Recognizing the limitations of current methods for treating MRSA and the urgent need for sustainable alternative drugs, researchers have turned their attention to identifying active molecules from unique microorganisms for further development into new antibiotics. Insects are widely distributed in nature, and their intestines provide a unique habitat for diverse microorganisms. These microorganisms are both numerous and varied [4]. To adapt to the complex natural environment, insects have developed a unique and efficient defense system through long-term evolution. The microorganisms within their bodies produce specialized metabolic compounds that resist the invasion of foreign pathogens, thereby protecting the host and enabling survival in challenging conditions. This demonstrates that insect-associated microorganisms possess the capacity to generate a diverse array of antibacterial substances, making them a valuable resource for discovering new antibiotics [5]. The number of active natural products isolated from the secondary metabolites of insect-associated microorganisms is increasing year by year. For example, natural products with diverse bioactive compounds have been isolated from the intestinal microorganisms of various insects, such as ants [6], locust [7], *Blattella germanica* [8], and *Copris tripartitus* [9]. These compounds serve as valuable candidates for the development of microbial drugs.

As a medicinal insect, *Blaps rynchopetera* is rich in bioactive compounds, including alkaloids, terpenoids, flavonoids, and phenolic derivatives, which exhibit antibacterial, anticancer, anti-inflammatory, antiviral, and antioxidant properties [10,11]. The Yunnan ethnic medicinal insect *B. rhynchopetera* harbors a rich diversity of microorganisms. In the initial stages, 108 fungal strains were isolated from the intestines of this insect. Among these fungi, *Fusarium oxysporum* BPF55 exhibited a significant inhibitory effect against MRSA, producing an inhibition zone measuring 14.9 ± 1.2 mm [12]. We further applied crystal violet staining to evaluate the anti-MRSA biofilm activity of secondary metabolites from *F. oxysporum* BPF55. The results showed that the secondary metabolites from this fungus exhibited inhibitory effects on MRSA biofilm formation. According to literature reports, *Fusarium* fungi can produce a variety of compounds with novel structures and significant biological activities, including alkaloids, terpenoids, quinones, polyketides, and others [13,14]. Studies have also shown that metabolites of *F. oxysporum* exhibit broad-spectrum antibacterial activity [15]. Lateropyrone, isolated from the endophytic fungus *Fusarium* sp. BZCB-CA of *Bothriospermum chinense*, exhibits inhibitory effects against MRSA and vancomycin-resistant *Enterococcus faecalis* [16]. The compound 2,4-di-tert-butylphenol, isolated from the endophytic fungus *F. oxysporum* TPL11 obtained from the perennial herb *Tradescantia pallida*, also exhibited antibacterial activity against MRSA [17]. Research on various *Fusarium* strains offers the opportunity to discover compounds that inhibit drug-resistant bacteria [18].

Currently, genome mining has become a powerful technology for exploring natural products [19]. and for altering metabolic product synthesis pathways through genetic modification [20]. It is particularly well-suited for discovering the diverse secondary metabolites found in endophytes [21]. The antiSMASH analysis tool has revolutionized the automated genomic mining of biosynthetic gene clusters (BGCs), significantly accelerating the discovery of new natural products [22]. To comprehensively analyze the secondary metabolic potential of microorganisms, it is essential to establish a practical and effective strategy for the bidirectional prediction of metabolite structure types that links genes to metabolism.

Given the critical role of biofilms in MRSA infectious diseases and the widespread emergence of multidrug-resistant strains, there is an urgent need to develop anti-MRSA agents capable of regulating biofilm formation. With ongoing research and breakthroughs, the interactions between chemical molecules and biomembranes have become increasingly well understood. However, no clinically approved drugs specifically targeting biofilms are currently available. This study aims to identify potential anti-MRSA biofilm compounds derived from insect-associated endophytes, offering an approach for discovering active compounds against MRSA infections. Based on this background, we employed whole-genome sequencing combined with Ultra-Performance Liquid Chromatography-Tandem Mass Spectrometry (UPLC-MS/MS) analysis to investigate the secondary metabolites of *F. oxysporum* BPF55. Additionally, we applied network pharmacology to explore its potential anti-MRSA biofilm active compounds. Our objective was to reveal the metabolic diversity of this strain and identify characteristic antibacterial compounds, thereby providing a reference for the development of natural product-based anti-MRSA drugs.

## 2. Materials and Methods

### 2.1. Culture of Fungi

The fungus was isolated from the intestinal tract of *B. rynchopetera* collected in Dali City, Yunnan Province. ITS sequencing and comparison with the NCBI database revealed a 100% sequence similarity to *F. oxysporum*. In previous studies, this strain was identified as *F. oxysporum* BPF55, with the accession number OP364039.1 [12]. The strain is preserved at the Yunnan Provincial Key Laboratory of Entomological Biopharmaceutical R&D. The fungus was inoculated onto PDA medium (Guangdong Huankai Biotechnology Co., Ltd., Guangzhou, China) and cultured at 28 °C for 5 days to promote growth. The growth morphology of the fungus on the PDA plate was photographed and documented. Subsequently, the mycelium was transferred to PDB medium (Guangdong Huankai Biotechnology Co., Ltd., Guangzhou, China), where it was cultured at 160 rpm. After incubation at a constant temperature of 28 °C for 7 days, the culture was subjected to high-speed centrifugation (Anhui Zhongke Zhongjia Scientific Instrument Co., Ltd., Hefei, China) at 4000 rpm for 10 min to collect the cells. The centrifuge tube was then rapidly frozen in liquid nitrogen and transferred to dry ice for preservation. Whole-genome sequencing was performed by Guangdong Magigene Biotechnology Co., Ltd (Guangzhou, China).

### 2.2. Fungal Genomic DNA Extraction and Preparation of DNA Library

Genomic DNA was extracted using a plant DNA extraction kit (Findrop Biosafety Technology Co., Ltd., Guangzhou, China) according to the manufacturer’s instructions. DNA integrity and purity were evaluated by 1% agarose gel electrophoresis. DNA concentration and purity were further quantified using a Qubit 4.0 fluorometer and a NanoDrop One spectrophotometer (Thermo Fisher Scientific, Waltham, MA, USA).

Sequencing libraries were prepared using the ALFA-SEQ DNA Library Prep Kit (Findrop Biosafety Technology Co., Ltd., Guangzhou, China) according to the manufacturer’s instructions [23]. The size of the library fragments was evaluated using the Qsep400 High-Throughput Nucleic Acid and Protein Analysis System (Houze Biological Technology Co., Hangzhou, China), and the library concentration was measured with a Qubit 4.0 fluorometer (Thermo Fisher Scientific, Waltham, MA, USA).

### 2.3. Genome Sequencing, Assembly and Gene Prediction

The genome of *F. oxysporum* BPF55 was sequenced using the Illumina NovaSeq×Plus platform (Illumina, Inc., San Diego, CA, USA), Trimmomatic v0.36 was used to filter low-quality reads [24], and the second-generation data were further assembled using SPAdes version 3.13.0 [25]. After filtering out contigs shorter than 500 bp, the final assembly was obtained. The quality and integrity of the assembled genome were assessed using Benchmarking Universal Single-Copy Orthologs (BUSCO v5.4.3) and Augustus annotation [26]. Genome composition prediction involves multiple complementary methods. Coding gene prediction of genomic sequences was performed using GeneMark-ES Suite version 4 (https://exon.gatech.edu/GeneMark/, accessed on 9 March 2026) [27]. tRNAscan-SE v2.0.11 was used for tRNA prediction [28], Barrnap (v0.9) for rRNA prediction, and Aragorn (v1.2.41) for tmRNA prediction. RepeatMasker v4.1.2 and Tandem Repeat Finder (TRF) v4.09 were employed to predict interspersed and tandem repeats, respectively.

### 2.4. ANI Calculation, Annotation and Biosynthetic Gene Cluster Analysis of the Genome

Average Nucleotide Identity (ANI) is one of the most reliable measures of genomic similarity between strains and holds significant potential in microbial taxonomy. The ANI calculator is available online at https://www.ezbiocloud.net/tools/ani accessed on 3 February 2026 [29]. The raw genomic data of this fungus were uploaded to the NCBI database to obtain a BioProject ID.

Functional annotation of protein sequences was performed using universal databases, including Gene Ontology (GO) [30], Kyoto Encyclopedia of Genes and Genomes (KEGG) [31], EuKaryotic Orthologous Groups (KOG) [32], and the Carbohydrate-Active Enzymes Database (CAZy) [33]. The complete genome annotation circular diagram of *F. oxysporum* BPF55 was generated using Circos version 0.69-8 [34].

AntiSMASH version 8.0.0 [35] was used to predict the secondary metabolite BGCs of *F. oxysporum* BPF55. By identifying, classifying, and comparing these BGCs, the fungus’s potential to synthesize secondary metabolites was thoroughly investigated.

### 2.5. Extraction of Secondary Metabolites from Fungi

The fungus *F. oxysporum* BPF55 was inoculated onto PDA medium and cultured in a biochemical incubator at 28 °C for 5 days. Its growth morphology was observed to confirm the absence of contamination by other microorganisms. Subsequently, mycelial plugs were transferred onto 20 PDA plates and cultured at 28 °C for 14 days. After cultivation, the mycelia and agar were quickly cut into small pieces and placed in an Erlenmeyer flask. The material was soaked and extracted three times with an organic solvent mixture of ethyl acetate, methanol, and glacial acetic acid in a ratio of 80:15:5, then concentrated under reduced pressure to obtain the crude extracts. Ultrapure water was added to the crude extracts to form a suspension, which was then transferred to a separatory funnel. An equal volume of ethyl acetate was added, and the extraction was performed three times. The combined ethyl acetate layers were evaporated to dryness to yield the ethyl acetate extract for subsequent analysis.

### 2.6. Evaluation of the Anti-Biofilm Activity of Crude Extracts Against MRSA

The microbroth dilution method was used to evaluate the anti-MRSA (ATCC 43300) activity of the crude extracts of *F. oxysporum* BPF55. After activation at 37 °C, MRSA was prepared into a bacterial suspension with a concentration of 1.0 × 10^6^ CFU/mL. The crude extracts and the positive control drug, vancomycin hydrochloride (Shanghai Maclin Biochemical Technology Co., Ltd., Shanghai, China), were initially dissolved in a small volume of DMSO (Rionlon BoHua Pharmaceutical & Chemical Co., Ltd., Tianjin, China), followed by the addition of sterile water to prepare a stock solution at 256 μg/mL. Subsequently, 100 μL of the crude extracts and an equal volume of bacterial suspension were added to a sterile 96-well plate and serially diluted to concentrations of 128, 64, 32, 16, 8, 4, 2, 1, 0.5, and 0.25 μg/mL, maintaining the DMSO content in the reaction system below 0.3%. The final volume in each well was 100 μL. A DMSO control group and a blank control group were included, with each group performed in triplicate. The plates were incubated at 37 °C for 24 h, after which bacterial growth was assessed by measuring the optical density at 600 nm using a multifunctional microplate reader (Varioskan™LUX, Thermo Fisher Scientific Inc., Shanghai, China). The minimum inhibitory concentration (MIC) of the crude extracts against MRSA was determined based on these results.

The MRSA (ATCC 43300) used in this study was purchased from Guangdong Microbial Culture Collection Center (GDMCC, Institute of Microbiology, Guangdong Province Academy of Sciences, Guangzhou, China), and previous Congo red experiments confirmed its strong biofilm formation ability. The inhibitory activity of crude extracts against MRSA biofilm formation was evaluated using crystal violet staining, as previously described [36]. A single MRSA colony was picked and inoculated into 5 mL of tryptic soy broth (TSB, Guangdong Huankai Biotechnology Co., Ltd., Guangzhou, China) medium for shaking culture at 37 °C and 160 rpm. The bacterial suspension was then diluted to approximately 1.0 × 10^6^ CFU/mL. Subsequently, 100 μL of this suspension was added to each well of a 96-well plate, followed by 100 μL of the crude extracts. The crude extracts were initially prepared at a stock concentration of 800 μg/mL, with serial dilutions made to final concentrations of 400, 200, 100, and 50 μg/mL. The experiment included four groups: the experimental group, containing 100 μL each of bacterial suspension and crude extracts; the positive control group, consisting of 100 μL bacterial suspension and 100 μL vancomycin at a final concentration of 12.5 μg/mL; the negative control group, containing 200 μL bacterial suspension only; and three replicate wells per group. The 96-well plate was incubated statically at 37 °C for 24 h. After incubation, the supernatant was discarded, and the wells were washed three times with an equal volume of sterile phosphate-buffered saline (PBS, Beijing Lanjike Technology Co., Ltd., Beijing, China). Then, 200 μL of methanol was added for 15 min and subsequently removed. The plate was dried at 37 °C for 30 min before adding 100 μL of 0.1% crystal violet (Beijing Solaibao Technology Co., Ltd., Beijing, China) to each well, which was allowed to stain for 10 min. The plate was gently rinsed with running water to remove excess stain. The stained biofilm at the bottom of each well was dissolved with 200 μL of 33% glacial acetic acid, and the optical density (OD) was measured at 595 nm using a microplate reader. Absorbance values were used to calculate relative percentages between the experimental and control groups. Data were plotted and analyzed using GraphPad Prism 8.0 software.

### 2.7. UPLC-MS/MS Testing and Data Analysis

The ethyl acetate extract of *F. oxysporum* BPF55 was analyzed using an Ultimate 3000 UPLC system (Thermo Fisher Scientific Inc., Waltham, MA, USA). Chromatographic separation was performed with an ACQUITY UPLC^®^ HSS T3 column (2.1 × 100 mm, 1.8 μm; Waters, Milford, MA, USA), maintained at 40 °C. The flow rate and injection volume were set at 0.3 mL/min and 2 μL, respectively. For LC-ESI (+)-MS analysis, the mobile phases consisted of 0.1% formic acid in acetonitrile (*v*/*v*) as mobile phase B2 and 0.1% formic acid in water (*v*/*v*) as mobile phase A2. Separation was performed using the following gradient: 0–1 min, 10% B2; 1–5 min, 10–98% B2; 5–6.5 min, 98% B2; 6.5–6.6 min, 98%–10% B2; and 6.6–8 min, 10% B2. For LC-ESI (−)-MS analysis, the analytes were separated using acetonitrile (B3) and 5 mM ammonium formate (A3) as mobile phases. Separation was performed using the following gradient: 0–1 min, 10% B3; 1–5 min, 10–98% B3; 5–6.5 min, 98% B3; 6.5~6.6 min, 98%~10% B3; 6.6~8 min, 10% B3 [37].

Mass spectrometric detection of metabolites was performed using a Q Exactive Focus (Thermo Fisher Scientific Inc., Waltham, MA, USA) equipped with an ESI ion source. Simultaneous MS1 and MS/MS acquisition was conducted in Full MS-ddMS2 mode (data-dependent MS/MS). The parameters were as follows: sheath gas pressure, 40 arb; auxiliary gas flow, 10 arb; spray voltage, 3.50 kV for ESI (+) and −2.50 kV for ESI (−); capillary temperature, 325 °C; MS1 scan range, *m*/*z* 150–2000; MS1 resolving power, 70,000 FWHM; number of data-dependent scans per cycle, 3; MS/MS resolving power, 17,500 FWHM; normalized collision energy, 30 eV; dynamic exclusion time, automatic [38].

The raw data were first converted to mzXML format using MSConvert from the ProteoWizard software package (v3.0.8789) [39] and processed with R XCMS (v3.12.0) [40] for feature detection, retention time correction, and alignment. The key parameter settings were as follows: bw = 2, ppm = 15, peakwidth = c (5, 30), mzdiff = 0.01, and method = centWave. Subsequently, the data were corrected using the area normalization method to eliminate systematic errors.

The metabolites were annotated by accurate mass measurements and MS/MS data, which were matched with HMDB (http://www.hmdb.ca) [41], massbank (http://www.massbank.jp/) [42], LipidMaps (http://www.lipidmaps.org) [43], mzcloud (https://www.mzcloud.org) [44], KEGG (https://www.genome.jp/kegg/) [45], accessed on 14 August 2025, and the metabolite database developed by Panomix Biomedical Tech Co., Ltd. (Shuzhou, China). The molecular weight of metabolites was determined based on the *m*/*z* (mass-to-charge ratio) of the parent ions in the MS data. The molecular formula was predicted using the adduct ion and then matched against the database. Simultaneously, the MS/MS data from the quantitative MS/MS dataset were compared with the fragment ions and other relevant information for each metabolite in the database to achieve MS/MS-based metabolite annotation.

### 2.8. Molecular Docking

To investigate the possible binding mode between metabolites from the fungus *F. oxysporum* BPF55 and proteins involved in MRSA biofilm formation, we performed molecular docking using AutoDock Tools 1.5.7 software [46]. During the docking analysis, all protein and ligand files were converted to PDBQT format, with all water molecules removed and polar hydrogen atoms added. The grid box was centered over the functional domain of each protein, allowing unrestricted molecular movement. Visualization of the docking results was conducted using PyMOL 2.6 and Discovery Studio 2021 software. The molecular structures of the compounds were obtained from the PubChem compound database (https://pubchem.ncbi.nlm.nih.gov/) and converted to mol2 format using Chem3D 22.2.0.3300 software. The protein structures of the target proteins SarA (PDB ID: 2FRH) and AgrA (PDB ID: 4G4K) were obtained from the RCSB Protein Data Bank.

## 3. Results

### 3.1. The Growth Morphology of F. oxysporum BPF55 and Its Genome Sequencing, Assembly and Genomic Features

**Figure 1:**
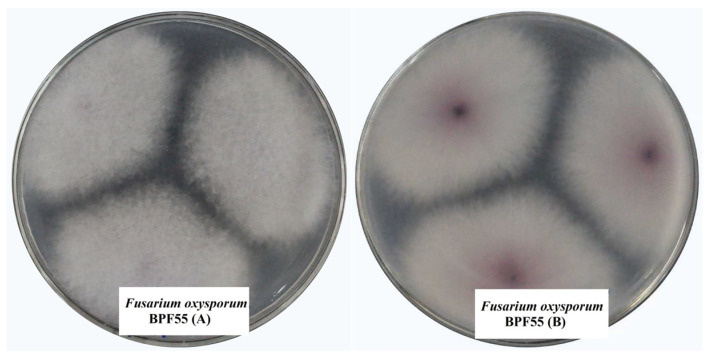
The growth morphology of *F. oxysporum* BPF55 on the PDA medium. (**A**) depicts the obverse side, and (**B**) shows the reverse side.

The growth morphology of *F. oxysporum* BPF55 on the PDA plate, including both sides, is shown in Figure 1.

Using Illumina NovaSeq×Plus sequencing technology, the de novo assembly of the *F. oxysporum* BPF55 genome was completed. As shown in Table 1, the fungal genome assembly comprises 712 contigs, with a total length of 50,097,681 bp. The largest contig measures 1,600,307 bp, and the GC content is 47.36%. A total of 16,507 genes were predicted, with a combined length of 23,884,490 bp and an average gene length of 1446.93 bp.

**Figure 2:**
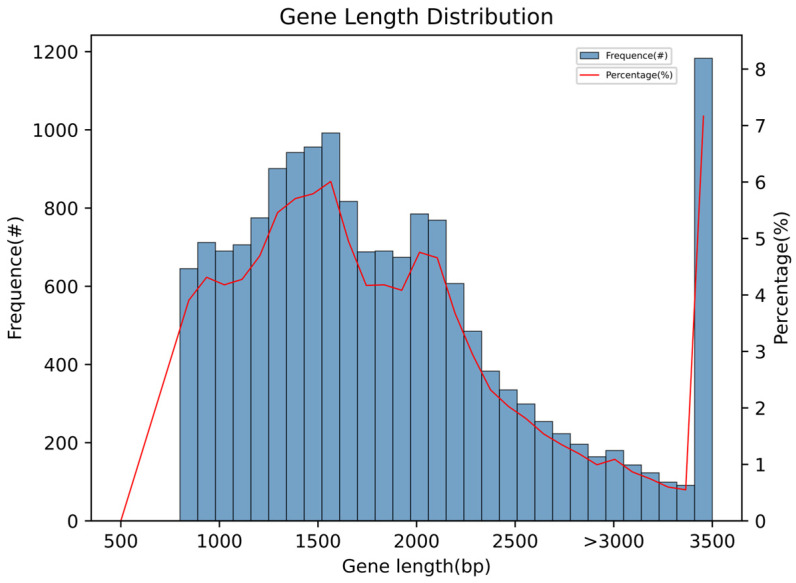
Gene length distribution of *F. oxysporum* BPF55. The horizontal axis represents length intervals, while the vertical axis indicates the number of genes within each interval. Due to the wide range of sequences longer than 3500 bp, these are grouped together.

**Figure 3:**
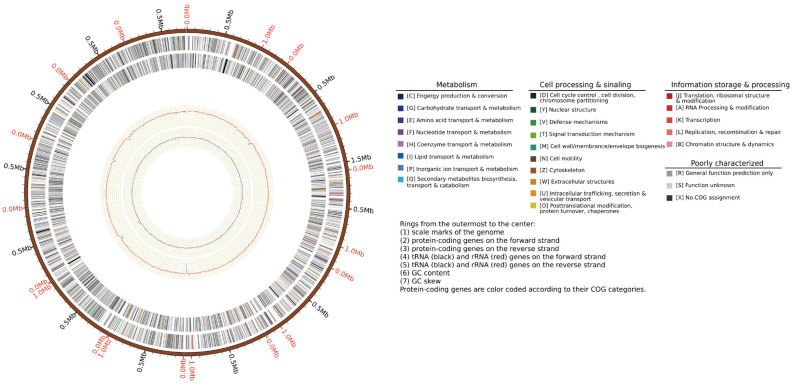
Genome annotation circular diagram of *F. oxysporum* BPF55. The tracks from the outside to the inside were the gene information in turn as scale marks of the genome; protein-coding genes on the forward strand; protein-coding genes on the reverse strand; tRNA (black) and rRNA (red) genes on the forward strand; tRNA (black) and rRNA (red) genes on the reverse strand; GC content; GC skew. Protein-coding genes are color-coded according to their KOG categories.

The gene length distribution map indicates that the largest number of genes fall within the 1200–1500 bp range, while the fewest genes are between 2500 and 3500 bp in length, as shown in Figure 2. Genome completeness was assessed using BUSCO v5.4.3, revealing 98.9% completeness, including 98.4% single-copy and 0.5% duplicated genes. Additionally, 0.4% of genes were fragmented, and 0.7% were missing. The genome annotation circular diagram of *F. oxysporum* BPF55 is shown in Figure 3.

Repeat sequences are widespread in the genomes of eukaryotes, containing a substantial amount of genetic information and playing a crucial role in gene regulatory networks. Table 2 presents the repeat sequence statistics for *F. oxysporum* BPF55. Within the genome, the total length of 12,411 direct repeats (DRs) is 14,589 bp, accounting for 0.03% of the genome. These repeats include long terminal repeats (LTRs), long interspersed nuclear elements (LINEs), short interspersed nuclear elements (SINEs), and DNA transposons. This analysis was performed using RepeatMasker (v4.1.2). Additionally, one unidentified sequence was predicted.

### 3.2. ANI Computational Analysis of the Genome

Previous research has identified BPF55 in molecular biology studies. By extracting its genomic DNA, amplifying the ITS rRNA region, and performing a BLAST + 2.12.0 comparison with data from GenBank, we determined that BPF55 belongs to the species *F. oxysporum* [12]. Based on whole-genome sequencing results, the average nucleotide identity (ANI) between the genome of *F. oxysporum* BPF55 and the reference genome *F. oxysporum* Fo47 (GCA_013085055.1) is 96.76% (Table 3), which exceeds the 95% threshold. This provides clear genomic evidence supporting the taxonomic classification of BPF55 as *F. oxysporum*. The raw genomic sequencing data of this fungal strain have been deposited in NCBI and assigned the BioProject ID PRJNA1370335.

### 3.3. Gene Functional Annotation

**Figure 4:**
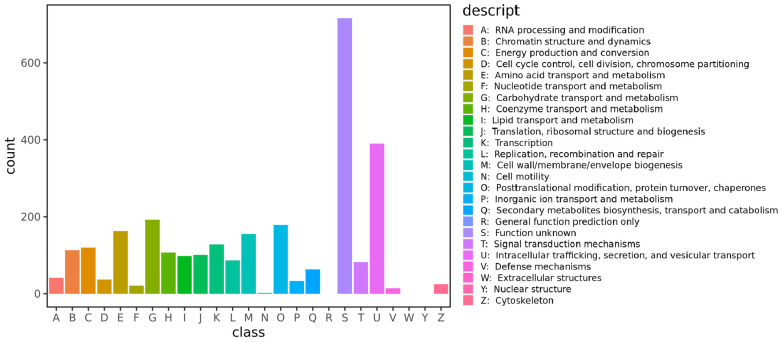
KOG function classification of the consensus sequence from *F. oxysporum* BPF55.

The *F. oxysporum* BPF55 genome contains 16,507 genes, most of which are annotated in the GO, KOG, and KEGG databases. Among these, 2867 genes are annotated in the KOG database, representing 17.37% of the total gene count (Table S1). The largest KOG category is “Function unknown”, with 716 genes, followed by “ Intracellular trafficking, secretion, and vesicular transport” (390), “Carbohydrate transport and metabolism” (192), “Posttranslational modification, protein turnover, chaperones” (179), “Amino acid transport and metabolism” (163), and “Cell wall/membrane/envelope biogenesis” (155) (Figure 4).

**Figure 5:**
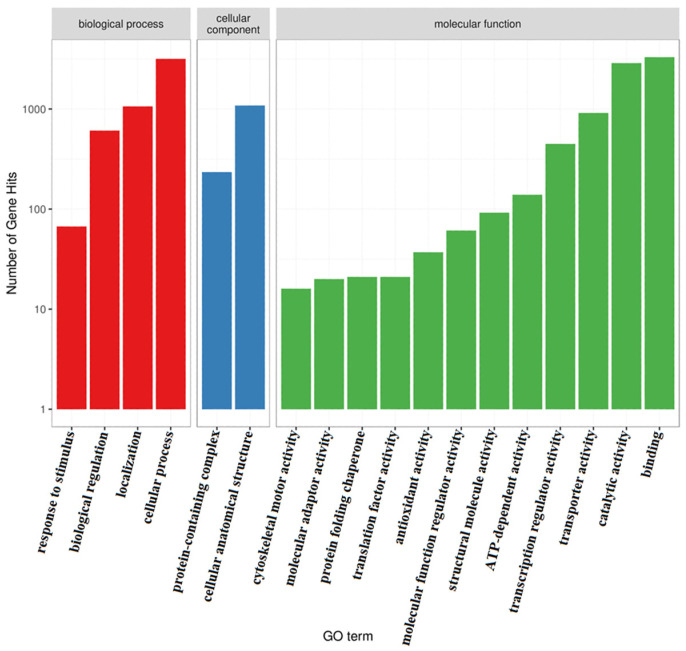
GO annotation of the genome of *F. oxysporum* BPF55.

The GO enrichment analysis revealed that 14,159 genes (85.78% of the genome) were annotated into three functional categories: biological processes, cellular components, and molecular functions (Table S1). Among the biological processes, 3167 genes were involved in cellular processes, followed by “localization”, “biological regulation”, and “response to stimulus”. Within the cellular components category, 1083 and 234 genes were associated with “cellular anatomical structure” and “protein-containing complex”, respectively. Regarding molecular functions, 3296, 2875, 913, 448, 139, 92, 61, 37, 21, 21, 20, and 16 genes participated in “binding”, “catalytic activity”, “transporter activity”, “transcription regulator activity”, “ATP-dependent activity”, “structural molecule activity”, “molecular function regulator activity”, “antioxidant activity” “protein folding chaperone”, “translation factor activity”, “molecular adaptor activity”, and “cytoskeletal motor activity”, respectively (Figure 5).

**Figure 6:**
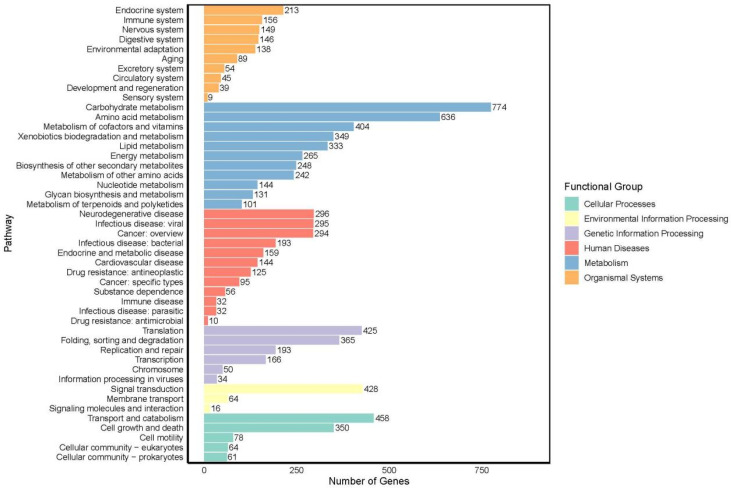
KEGG pathway annotation of the genome of *F. oxysporum* BPF55.

KEGG enrichment analysis revealed that 11,207 genes were annotated in the KEGG database (Table S1), accounting for 67.89% of the genome. The top five pathways with the highest number of genes are carbohydrate metabolism (774), amino acid metabolism (636), transport and catabolism (458), signal transduction (428), and translation (425) (Figure 6).

### 3.4. Carbohydrate-Active Enzymes

**Figure 7:**
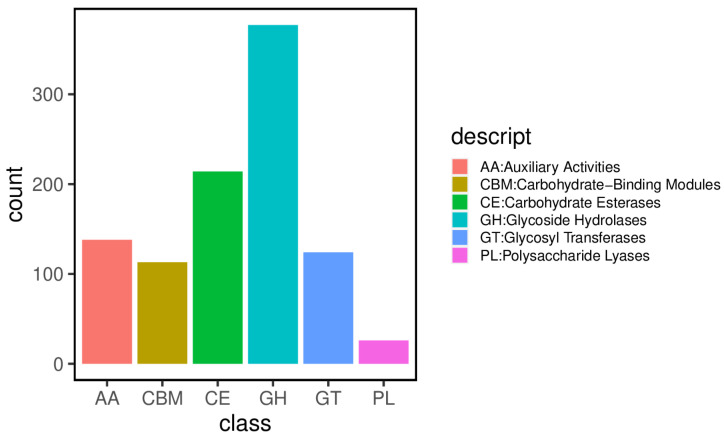
Distribution of carbohydrate-active enzyme (CAZyme) families.

The study utilized hmmscan, a tool linked to the Carbohydrate-Active Enzymes Database (CAZy), to compare the non-redundant gene set against the CAZy database and obtain corresponding carbohydrate-active enzyme annotations (Table S1). The results identified a total of 992 genes classified into six distinct CAZyme families. The most abundant enzyme family was Glycoside Hydrolases (GH), comprising 377 CAZyme-coding genes. The second most abundant family was carbohydrate esterases (CEs), with 214 genes. Additionally, 138 genes encoded auxiliary activities (AAs), and 124 genes were associated with glycosyl transferases (GTs). Furthermore, 113 genes encoded carbohydrate-binding modules (CBMs), and 26 genes encoded polysaccharide lyases (PLs) within the genome (Figure 7). CAZyme-coding genes are associated with diverse enzymatic activities. Among these enzymes, glycoside hydrolases (GHs), which constitute the largest proportion of coding genes, are responsible for degrading complex polysaccharides such as cellulose, hemicellulose, and lignin. This degradation process not only provides fungi with carbon sources and energy but also produces degradation products (such as monosaccharides and oligosaccharides) that can serve as signaling molecules or precursors to induce or regulate the expression of secondary metabolite biosynthesis gene clusters. Fungal CAZymes degrade carbohydrates, supplying energy and precursors essential for synthesizing fungal secondary metabolites. Although fungal CAZymes do not directly synthesize these secondary metabolites, they influence their production and accumulation by regulating energy metabolism and providing precursor substances.

### 3.5. Biosynthetic Potential of Secondary Metabolites

AntiSMASH version 8.0.0 was used to analyze the genome of *F. oxysporum* BPF55. A total of 56 BGCs were identified (Table S2). These gene clusters represent diverse biosynthetic pathways, including terpene-like compound synthesis gene clusters (15 clusters), which are the most abundant, and followed by non-ribosomal peptide synthase-like (NRPS-like) gene clusters (8 clusters); non-ribosomal peptide synthase (NRPS) gene clusters (5 clusters); type I polyketide synthase (T1PKS) gene clustaers (5 clusters); terpene precursor-type gene clusters (3 clusters); betalactone synthesis gene clusters (2 clusters); and cytokinin class synthesis gene clusters (2 clusters). The clusters also include several hybrid gene clusters, such as combined non-ribosomal polypeptide synthase and type I polyketide synthase (NRPS, T1PKS) gene clusters (2 clusters); NRPS and terpene gene clusters (2 clusters); combined T1PKS and NRPS-like (T1PKS, NRPS-like) gene clusters (2 clusters); combined NRPS and T1PKS gene clusters (NRPS, T1PKS) (2 clusters); and one type III polyketide synthase (T3PKS) gene cluster. The remaining seven gene clusters are predicted to be “NRPS, isocyanide-nrp”, “terpene, T1PKS, NRPS”, “NRPS, T1PKS, indole”, “NRP-metallophore, NRPS”, isocyanid, CDPS, and phosphonate synthetic gene clusters. Among them, 10 biosynthetic gene clusters exhibit high similarity to those of known compounds, including bikaverin, gibepyrone-A, depudecin, koraiol, clavaric acid, choline, oxyjavanicin, ACT-Toxin II, α-acorenol, and fosfonochlorin. Two biosynthetic gene clusters show moderate similarity to gene clusters encoding the known compounds equisetin and fusaric acid. Six biosynthetic gene clusters are similar to those of known compounds such as fusaridione A, squalestatin S1, gibberellin, alternapyrone, and beauvericin (with two BGCs). Additionally, several gene clusters exhibit low similarity, and 38 gene clusters do not match any known biosynthetic gene clusters, marked as “—”. These orphan gene clusters account for 67.9% of the total, indicating that the fungus harbors a substantial number of unidentified natural products.

### 3.6. Results of the Anti-Biofilm Activity of Crude Extracts Against MRSA

The crude extracts of *F. oxysporum* BPF55 were tested for anti-MRSA activity, with vancomycin hydrochloride serving as the positive control. The results showed that the MIC of vancomycin was 2 μg/mL, while the MIC of the crude extracts was 16 μg/mL, indicating that the crude extracts exhibited bactericidal activity.

**Figure 8:**
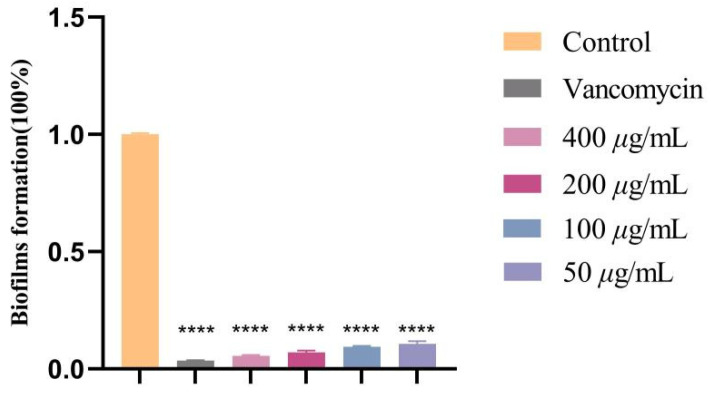
Effect of secondary metabolites on MRSA biofilm formation rate (Data expressed as means of three independent experiments. Error bars indicate standard errors of the means compared to control, statistically significant data, **** *p* < 0.0001).

To further investigate the anti-biofilm activity of the crude extracts produced by the fungus *F. oxysporum* BPF55, the in vitro inhibitory activity of various concentrations of crude extracts against MRSA biofilms was evaluated using crystal violet staining. The results demonstrated that, compared to the negative control group, both the positive control vancomycin and the crude extracts treatments significantly reduced biofilm formation (**** *p* < 0.0001). Furthermore, the rate of biofilm formation decreased in a dose-dependent manner with increasing metabolite concentration. The results showed that the concentration of vancomycin was 12.5 µg/mL, with a biofilm formation rate of only 3.61 ± 0.12%. When the concentration of the crude extracts was 400 µg/mL, the biofilm formation rate was 5.59 ± 0.36%. These findings indicate that the crude extracts of *F. oxysporum* BPF55 effectively inhibit MRSA biofilm formation (Figure 8).

### 3.7. UPLC-MS/MS Analysis of Secondary Metabolites

**Figure 9:**
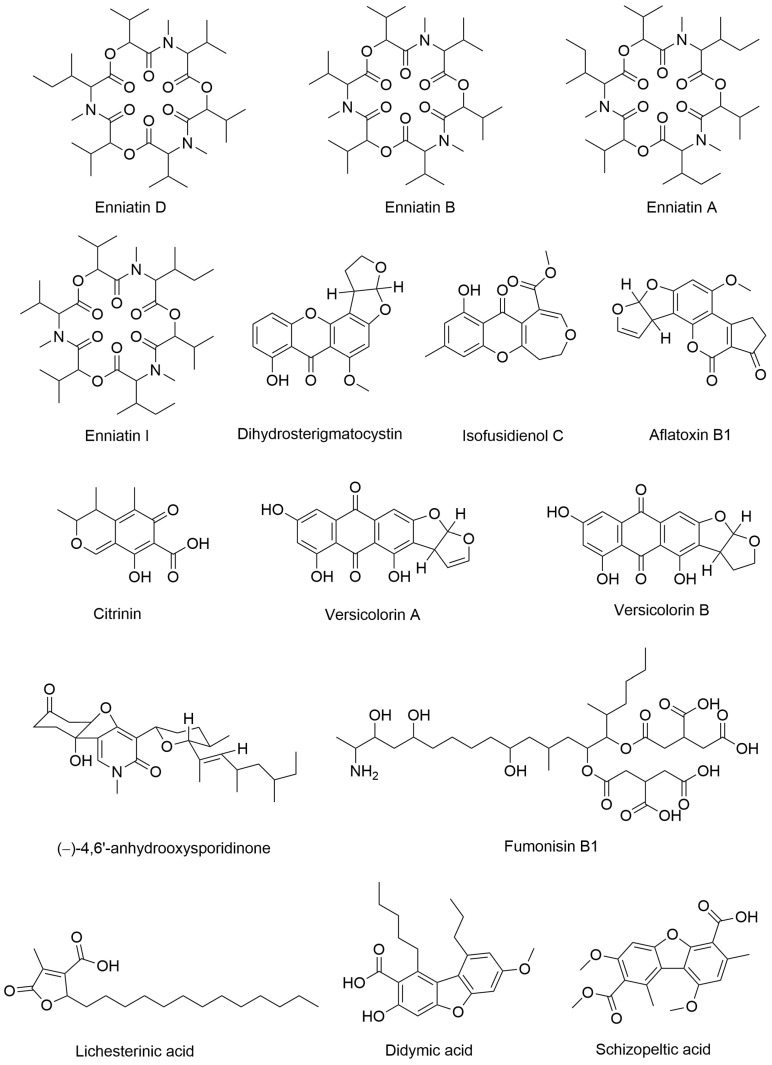
15 compounds annotated in the ethyl acetate extract.

In this study, the UPLC-MS/MS method was employed to qualitatively analyze the ethyl acetate extract of this strain. Total ion chromatograms were obtained in both positive and negative ion modes, as shown in Figure S1. The detected secondary metabolite peaks are presented in Figures S2 and S3, respectively. A total of 15 compounds were annotated from the ethyl acetate extract (Figure 9). According to literature reports, nine of these compounds were derived from fungi and exhibited antibacterial activity. The molecular formulas, mass spectra, and activity information of the annotated compounds are summarized in Table 4. Compounds with no reported relevant activity are indicated by “—”.

### 3.8. Molecular Docking and Analysis of Compounds with Core Proteins Involved in MRSA Biofilm Formation

This study employed AutoDockTools software to perform molecular docking of compounds annotated in the ethyl acetate extract of *F. oxysporum* BPF55 with SarA and AgrA—two key proteins involved in MRSA biofilm formation—to assess the affinity of these compounds for their respective targets. Fifteen compounds were docked to the two proteins (SarA, PDB ID: 2FRH, resolution 2.50 Å; AgrA, PDB ID: 4G4K, resolution 1.52 Å). Docking provided the binding conformations and energies of the compounds with these proteins. According to binding energy evaluation criteria, docking scores below −4.25 kcal·mol^−1^ indicate basic binding activity, scores around −5 kcal·mol^−1^ indicate good binding activity, and scores below −7 kcal·mol^−1^ indicate high affinity. The docking binding energies are presented in Table 5. The data show that the binding energies of all 15 compounds with the two targets are less than −5 kcal·mol^−1^, indicating that the ligands and receptors can form stable complexes. Furthermore, lower binding energies correspond to more stable interactions between the active compounds and the core targets.

**Figure 10:**
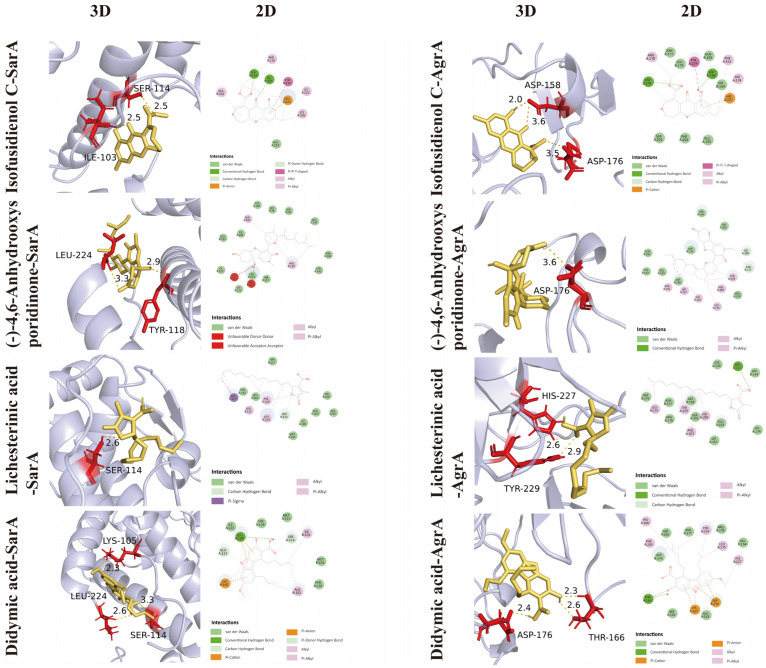
Molecular docking analysis: Two-dimensional (2D) and three-dimensional (3D) representations of the interaction patterns of four compounds with SarA and AgrA. Left panel: interactions between the four compounds and SarA of MRSA. Right panel: interactions between the four compounds and AgrA of MRSA.

Molecular docking analysis revealed that the 15 compounds annotated in the fungal ethyl acetate metabolites can interact with the SarA and AgrA proteins of MRSA. Based on the activity and toxicity profiles of the compounds, four bioactive compounds with distinct structures were selected for kinetic simulation analysis. The complexes of the four compounds with SarA—namely SarA-isofusidienol C, SarA-(−)-4,6′-anhydrooxysporidinone, SarA-lichesterinic acid, and SarA-didymic acid—as well as the complexes with AgrA—namely AgrA-isofusidienol C, AgrA-(−)-4,6′-anhydrooxysporidinone, AgrA-lichesterinic acid, and AgrA-didymic acid—were selected for visualization analysis. The binding energies of these four compounds to both SarA and AgrA were all lower than −5 kcal·mol^−1^, indicating stable binding (Table 6). The docking results of these four compounds with the active sites of SarA and AgrA proteins are shown in Figure 10. Notably, (−)-4,6′-anhydrooxysporidinone interacts with the active site of AgrA with a binding energy of −8.9 kcal·mol^−1^. The binding affinity of these compounds to SarA and AgrA suggests their potential efficacy against MRSA biofilms.

## 4. Discussion

The formation of biofilms significantly contributes to drug resistance in MRSA. Current research highlights the potential of natural anti-biofilm agents to prevent biofilm formation and eradicate existing biofilms by inhibiting the expression of bacterial virulence factors [62]. Notably, these agents possess unique properties that do not impede bacterial metabolic growth, thereby reducing the likelihood of developing drug resistance [63]. As a distinctive class of microorganisms, insect endophytes produce a diverse array of secondary metabolites, serving as an important source for discovering lead compounds. In this study, we employed a combination of whole-genome sequencing and UPLC-MS/MS to characterize the fungus *F. oxysporum* BPF55 and its ability to produce secondary metabolites. Additionally, molecular docking was used to further explore its potential metabolites with anti-MRSA biofilm activity.

Studies have shown that the average nucleotide identity (ANI) between the fungus BPF55 and the *F. oxysporum* strain Fo47 is 96.8%, confirming that the fungus strain belongs to *F. oxysporum* and has been designated *F. oxysporum* BPF55. After uploading the raw genomic data of this strain to NCBI, the associated BioProject number was assigned as PRJNA1370335. Through whole-genome sequencing and bioinformatics analysis, the genome size of *F. oxysporum* BPF55 was determined to be 1,600,307 bp, with a GC content of 47.36%, and it contains 16,507 coding genes. The predicted protein sequences of *F. oxysporum* BPF55 genes were compared against the KOG, GO, and KEGG functional databases, and gene functions were annotated based on amino acid sequence similarity. KOG classification results revealed that many proteins were annotated, indicating that their specific roles remain to be elucidated. GO enrichment analysis showed that the majority of annotated genes are involved in cellular processes. KEGG enrichment analysis indicated that genes related to carbohydrate metabolism and amino acid metabolism pathways were highly represented, suggesting that the fungus can catabolize proteins and carbohydrates from the environment to generate energy for survival. Additionally, a large number of CAZyme-coding genes, particularly glycoside hydrolases (GH) and carbohydrate esterases (CE), were identified through the CAZy database, indicating that this fungal strain is proficient at degrading complex carbohydrates.

AntiSMASH predicts 56 BGCs in *F. oxysporum* BPF55, which are involved in the synthesis of various natural products, including terpenes, non-ribosomal peptides, and polyketides. Similarity analysis with reported gene clusters revealed that 10 of these BGCs closely resemble those associated with known compounds, primarily synthesizing terpenes, non-ribosomal peptides, polyketides, and other natural products. Among these potential secondary metabolites, bikaverin exhibits antibacterial and antitumor activities [64]. Depudecin is a metabolite containing two epoxide groups that exhibits anti-angiogenic activity in vivo [65]. Clavaric acid is a triterpene compound known for its antitumor activity [66]. Fosfonochlorin is an antibiotic with spheroplast-forming activity [67]. The two biosynthetic gene clusters exhibit moderate similarity to the gene clusters responsible for producing the known compounds fusaric acid and equisetin. Fusaric acid demonstrated significant antibacterial activity against *S. aureus*, with a MIC of 1.30 µg/mL [68]. Equisetin is derived from the marine fungus *Fusarium* sp. 152 and is known for its potent antibacterial activity against Gram-positive bacteria, exhibiting a MIC of 1 μg/mL against MRSA [69]. The antibacterial mechanism of this compound is achieved by inhibiting bacterial acetyl-CoA carboxylase [70]. Recently, researchers have overcome solubility limitations and enhanced the clinical applicability of equisetin, making it a promising antibacterial treatment [71]. Beauvericin has been shown to effectively enhance the inhibitory effect of oxacillin against MRSA [72]. Additionally, *F. oxysporum* BPF55 contains 67.9% orphan gene clusters, which exhibit low similarity to previously reported gene clusters and may be responsible for synthesizing novel secondary metabolites.

Through UPLC-MS/MS data analysis, we annotated 15 compounds from *F. oxysporum* BPF55. Among these, enniatin A exhibited inhibitory effects against MRSA and was more active than enniatin B, suggesting that the presence of isopropyl groups reduces the ability of enniatin peptides to insert into bacterial lipids [48]. Enniatin D also inhibits *Staphylococcus aureus* [47]. Enniatin A, enniatin B, enniatin D, and enniatin I all belong to the class of cyclohexadepsipeptidic mycotoxins. Studies have shown that enniatin A and enniatin D exhibit certain toxicity to red blood cells [47]. The potential toxicity of enniatin B may be enhanced by co-occurrence with other enniatins or mycotoxins [73]. Aflatoxin B1 is the most carcinogenic natural mycotoxin and can cause adverse health effects in humans, including acute toxicity, immunosuppression, teratogenicity, mutagenicity, and carcinogenicity [49]. It also exhibits cytotoxic activity [50]. Versicolorin A and versicolorin B are biosynthetic precursors of aflatoxin B1, while Dihydrosterigmatocystin is a biosynthetic precursor of aflatoxin B2 [74]. Although literature reports that versicolorin B exhibits activity against MRSA at 6.25 µg/mL [57], both versicolorin A and versicolorin B have been shown to cause significant DNA damage [75]. Citrinin is a toxic polyketide known for its reproductive toxicity as well as teratogenic, nephrotoxic, hepatotoxic, and embryotoxic effects [51]. Citrinin also acts as an inhibitor of the quorum-sensing (QS) system in Pseudomonas aeruginosa. However, as a mycotoxin, it is also harmful to humans [52]. Despite its toxicity, citrinin has been reported to exhibit antimicrobial, anticancer, antioxidant, and neuroprotective activities [53]. Additionally, fumonisin B1 is a toxin, and long-term, high-dose exposure to fumonisin B1 increases cancer risk [59]. Isofusidienol C exhibits antibacterial activity against *Candida albicans* and is effective against both Gram-positive and Gram-negative bacteria [55]. The MIC of (−)-4,6′-anhydrooxysporidinone, isolated from the ethyl acetate extract of the fungus *F. oxysporum*, is 100 µg/mL [58]. Lichesterinic acid exhibits antioxidant and antihyperglycemic activities [60]. Didymic acid is active against *S. aureus*, with an MIC of 7.5 µg/mL [61]. Considering the potential toxicity or risks associated with compounds such as enniatins, aflatoxin, citrinin, and fumonisin B1, we selected four other bioactive compounds with different structural types for molecular docking: (−)-4,6′-anhydrooxysporidinone, isofusidienol C, lichesterinic acid, and didymic acid.

The formation of biofilms is a significant factor contributing to drug resistance in MRSA. The mechanisms underlying biofilm formation are complex and involve multiple regulatory factors and signaling pathways, such as quorum sensing (QS) and gene transcription [76]. The *Staphylococcus* regulatory systems responsible for controlling biofilm formation primarily include the accessory gene regulator (Agr) [77] and the *Staphylococcus* accessory gene regulator A (SarA) [78,79]. Agr coordinates intercellular communication and behavior based on cell density, a system critical for the efficient dissemination of MRSA from biofilms to adjacent tissues [80]. Specifically, the AgrA transcription factor regulates the QS response of MRSA and controls the production of hemolysin and other virulence factors [81]. SarA regulates biofilm formation through Agr-dependent pathways [82]. It binds to the Agr promoter, stimulates the transcription of RNA III, and triggers a secondary response that regulates downstream target genes, thereby promoting biofilm development [83]. SarA plays a key role in regulating the expression of multiple virulence genes. Disruption of SarA function can impair the transcription of essential messenger RNAs, consequently affecting pathogen virulence [84]. Studies have demonstrated that AgrA and SarA act synergistically to regulate the secretion of virulence factors and biofilm formation [85]. These proteins are key targets in the formation and development of MRSA biofilms [86] and are considered the most promising therapeutic targets for the development of anti-biofilm drugs.

In modern drug research and development, structure-based virtual screening technology provides robust support for identifying and designing multiple bioactive compounds targeting specific proteins. Computational simulation methods facilitate the prediction of interactions between known drug target proteins and their natural ligands. Given that AgrA and SarA are key target proteins involved in the formation and development of MRSA biofilms [84,87]. In this study, SarA and AgrA were selected for docking analysis of compounds. This study annotated 15 compounds in the *F. oxysporum* BPF55 metabolite that can stably bind to SarA and AgrA. Among these, four compounds—isofusidienol C, (−)-4,6′-anhydrooxysporidinone, lichesterinic acid, and didymic acid—exhibited low binding energies to both targets. Visual analysis revealed that these compounds interact with the active sites of both proteins, suggesting that the *F. oxysporum* BPF55 metabolite possesses an inhibitory mechanism against MRSA biofilm formation.

## 5. Conclusions

MRSA can enhance its antibiotic resistance through biofilm formation. This study conducted a comprehensive analysis of the genomics and secondary metabolites of the fungus *F. oxysporum* BPF55, revealing that it produces a diverse array of secondary metabolites with varied structures. Most of these metabolites demonstrated antibacterial activity, particularly by inhibiting MRSA biofilm formation. Molecular docking analysis showed that four metabolites could interact with the active sites of the SarA and AgrA proteins in MRSA. Overall, these findings suggest that *F. oxysporum* BPF55 is a promising strain with significant potential for development, and its active metabolites warrant further investigation as potential therapeutic agents against MRSA biofilms.

## Figures and Tables

**Table 1:** General features of genome assembly and prediction of *F. oxysporum* BPF55.

	General Features
contigs	712
Largest contig	1,600,307 bp
Total length	50,097,681 bp
GC (%)	47.36%
N50	502,217 bp
N75	188,023 bp
L50	31 bp
L75	68 bp
Number of predicted genes	16,507 bp
Total length of predicted genes	23,884,490 bp
Average length of predicted genes	1446.93 bp
GC percent (%)	51.46
Integrity	16,065

**Table 2:** Statistical analysis of interspersed repetitive sequences in *F. oxysporum* BPF55 Genome.

Repetitive Sequence	Number	Length Occupied	Percentage of Sequence (%)
SINEs	23	1240	0.00
LINEs	163	10,773	0.02
LTRs elements	2	95	0.00
DNA elements	37	2408	0.00
Unclassified	1	73	0.00
Total interspersed repeats		14,589	0.03

**Table 3:** Average nucleic acid similarity analysis of *F. oxysporum* BPF55.

Query ID	Reference ID	ANI	Mapped_Fragment	Query_Fragment	Taxon
BPF55	GCA 013085055.1	96.7617	2851	3103	*Fusarium oxysporum* Fo47 *chromosome*

**Table 4:** Compounds and functions in the ethyl acetate extract of *F. oxysporum* BPF55 detected by UPLC-MS/MS (Metabolites were annotated at MSI-level 2; *m*/*z*—ratio mass/charge detected; Rt—retention time).

Putative Metabolite	Formula	*m*/*z*	Rt	Precursor Type	Class	Function
Enniatin D	C_34_H_59_N_3_O_9_	654.4278	442.9	[M + H]^+^	cyclic peptide	antibacterial activity [47]
Enniatin B	C_33_H_57_N_3_O_9_	640.4102	424	[M + H]^+^	cyclic peptide	antibacterial activity [48]
Enniatin A	C_36_H_63_N_3_O_9_	682.4592	449.5	[M + H]^+^	cyclic peptide	antibacterial activity [48]
Enniatin I	C_35_H_61_N_3_O_9_	1335.8953	449.5	[2M + H]^+^	cyclic peptide	—
Aflatoxin B1	C_17_H_12_O_6_	311.0549	383.9	[M − H]^−^	polyketide	acute toxicity, immunosuppression, teratogenicity, mutagenicity, and carcinogenicity [49], cytotoxic activity [50]
Citrinin	C_13_H_14_O_5_	249.0766	252.7	[M − H]^−^	polyketide	reproductive toxicity and teratogenic, nephrotoxic, hepatotoxic and embryotoxic effects [51], quorum sensingquorum-sensing system inhibitors [52], antimicrobial, anticancer, antioxidant activities, neuroprotective effects [53]
Dihydrosterigmatocystin	C_18_H_14_O_6_	344.1115	216.2	[M + NH_4_]^+^	polyketide	antibacterial activity [54]
Isofusidienol C	C_16_H_14_O_6_	284.049	216.8	[M − NH_3_ − H]^−^	polyketide	antibacterial activity [55]
Versicolorin A	C_18_H_10_O_7_	397.0554	287.9	[M + CH_3_CO_2_]^−^	polyketide	mycotoxins, carcinogenicity [56]
Versicolorin B	C_18_H_12_O_7_	399.0718	281.1	[M + CH_3_CO_2_]^−^	polyketide	antibacterial activity [57]
(−)-4,6′-anhydrooxysporidinone	C_28_H_41_NO_5_	472.3055	406.4	[M + H]^+^	Polyketide, nitrogen-containing heterocyclic compound	antibacterial activity [58]
Fumonisin B1	C_34_H_59_NO_15_	678.399	397	[M − CO_2_ + H]^+^	polyketide	toxicity and carcinogenicity [59]
Lichesterinic acid	C_19_H_32_O_4_	325.2367	346.2	[M + H]^+^	fatty acid derivatives	radical scavenging activity [60]
Didymic acid	C_22_H_26_O_5_	369.1731	330.9	[M − H]^−^	Fatty acids, macrolides	antibacterial activity [61]
Schizopeltic acid	C_19_H_18_O_7_	357.0981	253.8	[M − H]^−^	polyketide	—

**Table 5:** Binding energy statistics for 15 compounds annotated in ethyl acetate extracts targeting core targets (kcal·mol^−1^).

Compounds	Targets	
	SarA	AgrA
Enniatin D	−6.4	−6.6
Enniatin B	−5.5	−6.1
Enniatin A	−7.3	−5.9
Enniatin I	−6.7	−5.8
Aflatoxin B1	−8.1	−8.9
Citrinin	−6.4	−7.6
Dihydrosterigmatocystin	−7.0	−8.1
Isofusidienol C	−7.3	−7.7
Versicolorin A	−8.3	−9.7
Versicolorin B	−8.1	−9.2
(−)-4,6′-anhydrooxysporidinone	−8.6	−8.9
Fumonisin B1	−5.9	−7.1
Lichesterinic acid	−5.1	−5.9
Didymic acid	−6.0	−7.3
Schizopeltic acid	−7.3	−7.7

**Table 6:** Molecular Docking Analysis Reveals the Binding Efficacy of Bioactive Compounds with SarA and AgrA of MRSA.

Receptor	Ligand	Key Residue /π Anion	π−π Tshape	Binding Energy (kcal/mol)
SarA	Isofusidienol C	SER A:114; ILE B:103; LEU A:224	TYRA:118	−7.3
	(−)-4,6′-anhydrooxysporidinone	LEU B: 224	—	−8.6
	Lichesterinic acid	SER B:114	—	−5.1
	Didymic acid	LEU A:224; SER A:114; GLU A:223; LYS B:105	—	−6.0
AgrA	Isofusidienol C	ASP B:176; ASP B:158; HIS A:227	TYRA:229	−7.7
	(−)-4,6′-anhydrooxysporidinone	HIS A: 200	—	−8.9
	Lichesterinic acid	HIS A:227	—	−5.9
	Didymic acid	THR A:166; LYS A:167; ASP B:158	—	−7.3

## Data Availability

The raw genomic sequencing data of *F. oxysporum* BPF55 have been deposited in NCBI and assigned the BioProject ID PRJNA1370335. This Whole Genome Shotgun project has been deposited at GenBank under the accession number JBVYOB000000000. Additionally, the GenBank accession number OP364039.1 is provided. All other data are included in the article’s Section 3.

## Supplementary Materials

The following supporting information can be downloaded at: https://www.mdpi.com/article/10.3390/jof12040236/s1, Table S1: Database Annotation of the *F. oxysporum* BPF55 Genome; Table S2: Prediction of Secondary Metabolite Gene Clusters in the *F. oxysporum* BPF55 Genome. Figure S1: Total Ion Chromatogram (TIC) of the ethyl acetate extract from *F. oxysporum* BPF55; Figure S2: The positive mode spectra peaks of the UPLC-MS/MS analysis for the ten metabolites produced by *F. oxysporum* BPF55; Figure S3: The negative mode spectra peaks of the UPLC-MS/MS analysis for the five metabolites produced by *F. oxysporum* BPF55.

## Abbreviations

The following abbreviations are used in this manuscript:

AMR	Antimicrobial resistance
Agr	Accessory gene regulator
ANI	Average Nucleotide Identity
CAZy	Carbohydrate-Active Enzymes Database
KOG	EuKaryotic Orthologous Groups
GO	Gene Ontology
BGCs	Biosynthetic gene clusters
DRs	Direct repeats
KEGG	Kyoto Encyclopedia of Genes and Genomes
LTRs	Long terminal repeats
LINEs	Long interspersed nuclear elements
MRSA	Methicillin-resistant *Staphylococcus aureus*
NRPS-like	Non-ribosomal peptide synthase-like
NRPS	Non-ribosomal peptide synthase
OD	Optical density
PBS	Phosphate-buffered saline
SINEs	Short interspersed nuclear elements
SarA	Staphylococcus accessory gene regulator A
T1PKS	Type I polyketide synthase
T3PKS	Type III polyketide synthase
TIC	Total Ion Chromatogram
TSB	Tryptic soy broth
UPLC-MS/MS	Ultra-Performance Liquid Chromatography-Tandem Mass Spectrometry
