# Supplementary material for: Genome and Secondary Metabolites Analysis of Fusarium oxysporum BPF55 Associated with Blaps rynchopetera and Its Anti-MRSA Biofilm Potential
Source: J Fungi (Basel). 2026 Mar 25;12(4):236. doi: 10.3390/jof12040236 (PMC13117560; doi:10.3390/jof12040236)
Supplement: Supplementary file 1 [file jof-12-00236-s001.zip › Figure S1 to Figure S3.pdf]

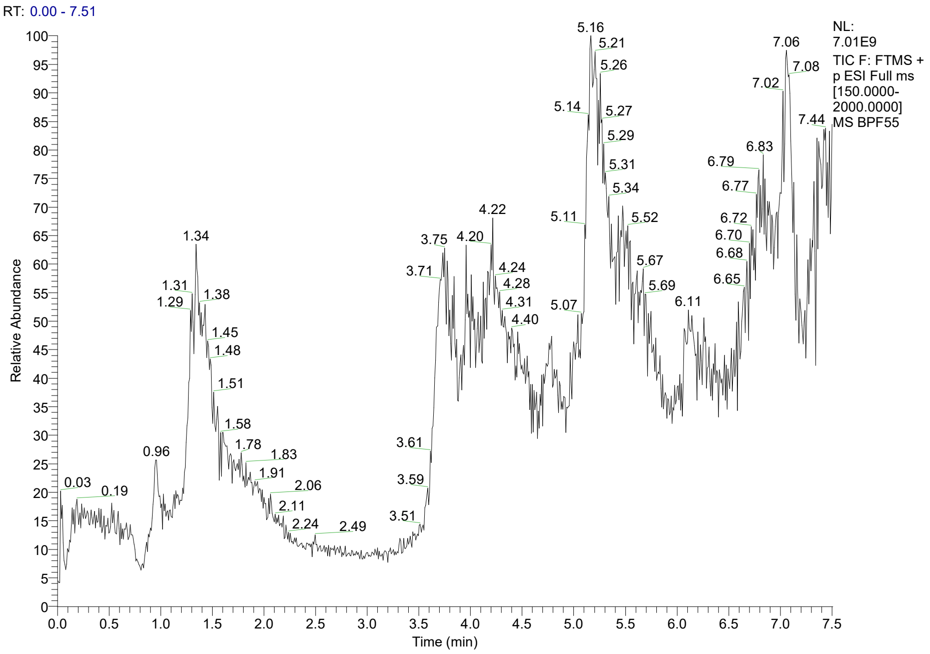

(a)

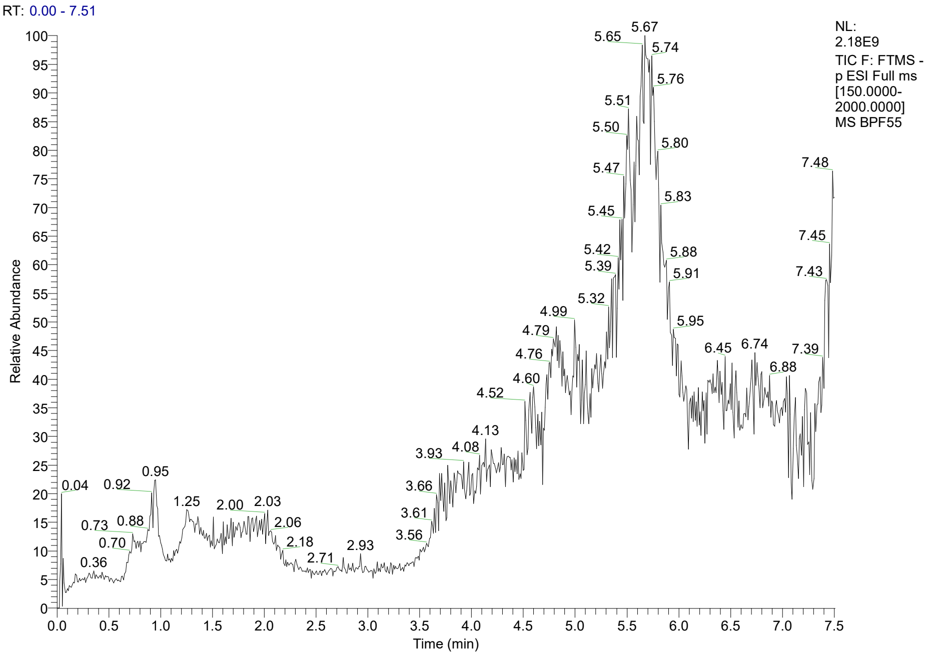

(b)

Figure S1. Total Ion Chromatogram (TIC) of the ethyl acetate extract from *F. oxysporum* BPF55. (a) TIC analyzed in positive ion mode; (b) TIC analyzed in negative ion mode.

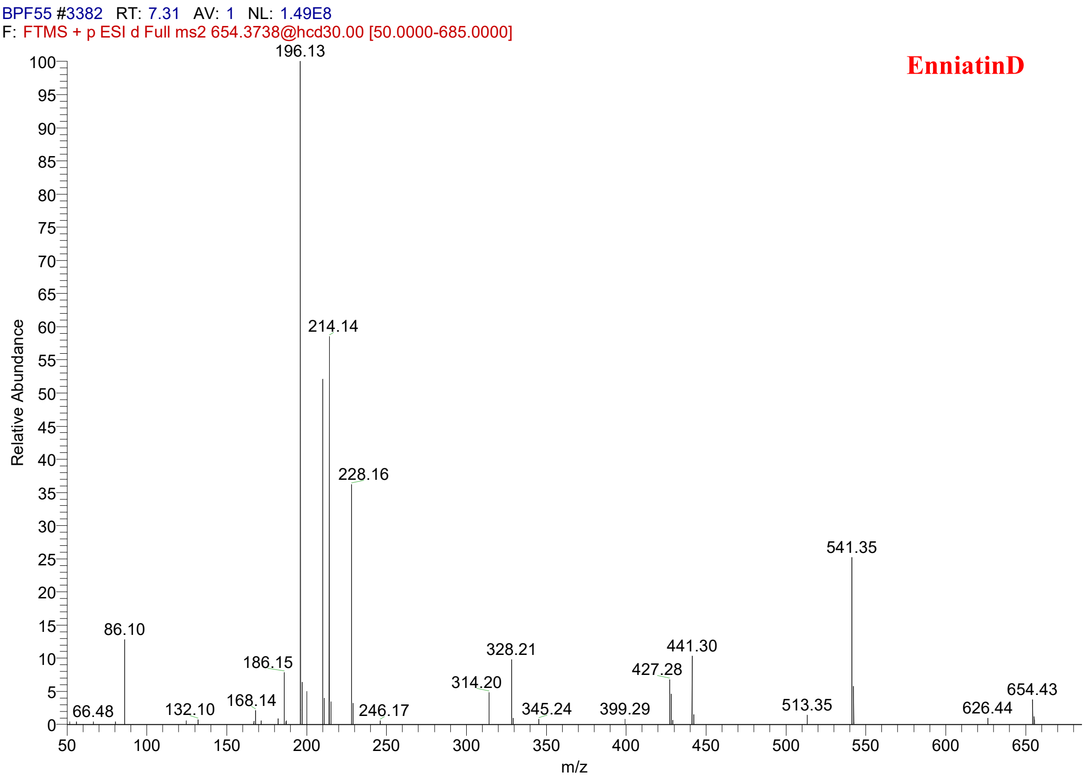
BPF55 #3382 RT: 7.31 AV: 1 NL: 1.49E8
F: FTMS + p ESI d Full ms2 654.3738@hcd30.00 [50.0000-685.0000]
EnniatinD
196.13
214.14
228.16
541.35
86.10
328.21
441.30
186.15
427.28
314.20
654.43
168.14
66.48
132.10
246.17
345.24
399.29
513.35
626.44
Relative Abundance
m/z

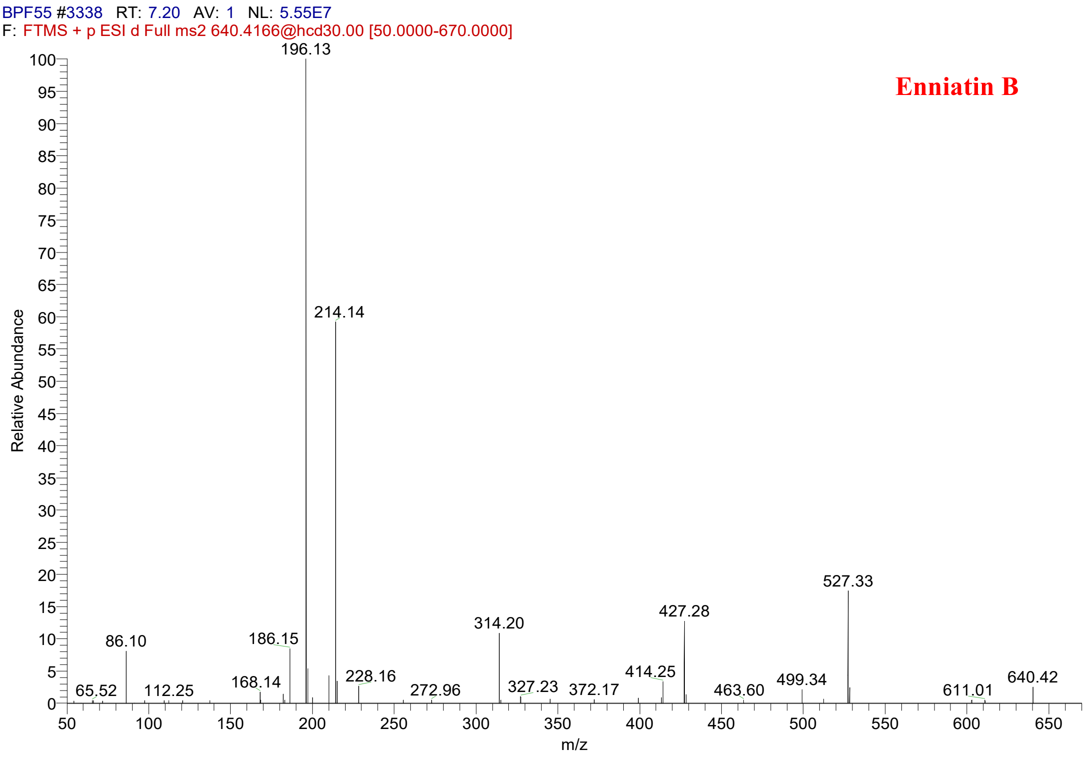
BPF55 #3338 RT: 7.20 AV: 1 NL: 5.55E7
F: FTMS + p ESI d Full ms2 640.4166@hcd30.00 [50.0000-670.0000]
Enniatin B
196.13
214.14
527.33
427.28
314.20
86.10
186.15
414.25
640.42
228.16
168.14
499.34
65.52
112.25
272.96
327.23
372.17
463.60
611.01
Relative Abundance
m/z

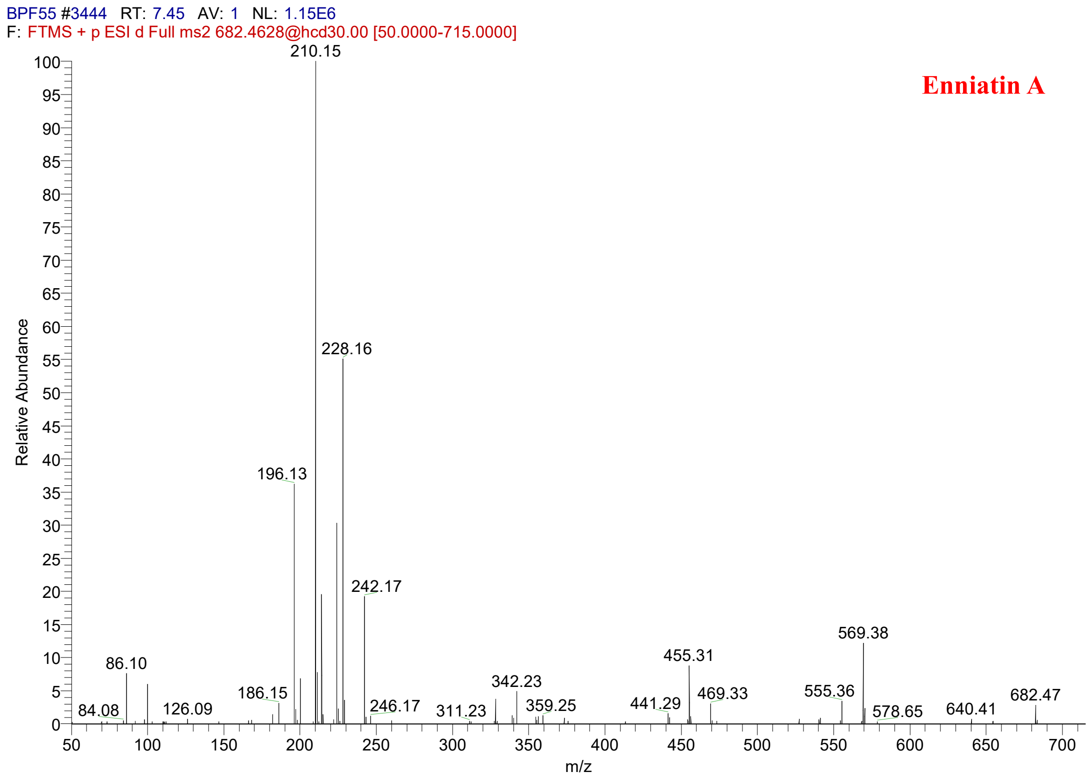
BPF55 #3444 RT: 7.45 AV: 1 NL: 1.15E6
F: FTMS + p ESI d Full ms2 682.4628@hcd30.00 [50.0000-715.0000]
Enniatin A
Relative Abundance
m/z
210.15
228.16
196.13
242.17
86.10
84.08
126.09
186.15
246.17
311.23
342.23
359.25
441.29
455.31
469.33
555.36
569.38
578.65
640.41
682.47

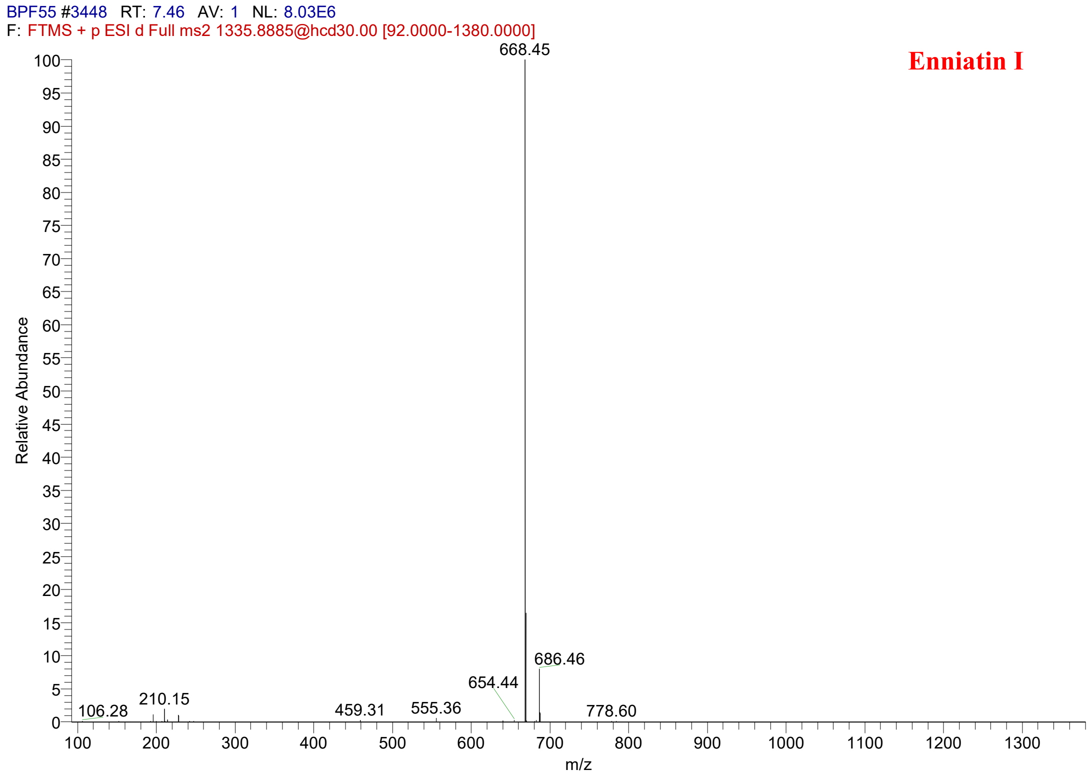
BPF55 #3448 RT: 7.46 AV: 1 NL: 8.03E6
F: FTMS + p ESI d Full ms2 1335.8885@hcd30.00 [92.0000-1380.0000]
Enniatin I
Relative Abundance
m/z
668.45
686.46
654.44
106.28
210.15
459.31
555.36
778.60

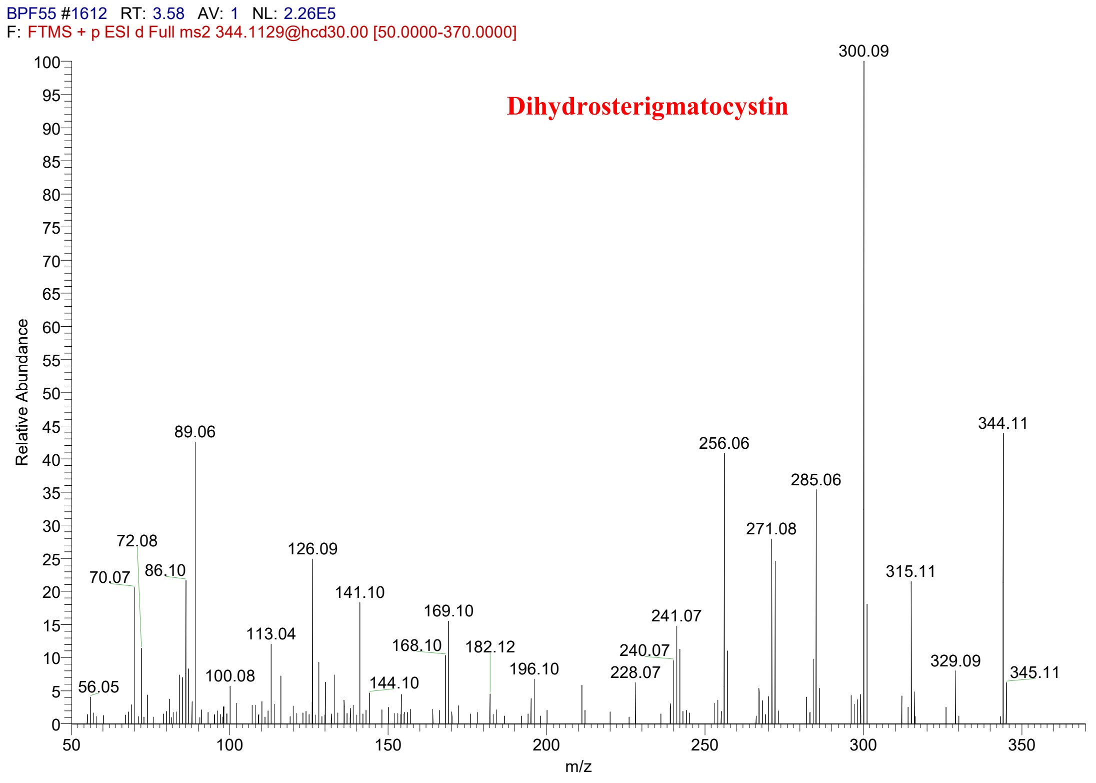
BPF55 #1612 RT: 3.58 AV: 1 NL: 2.26E5
F: FTMS + p ESI d Full ms2 344.1129@hcd30.00 [50.0000-370.0000]
Dihydrosterigmatocystin
Relative Abundance
m/z
300.09
344.11
89.06
256.06
285.06
271.08
72.08
126.09
86.10
315.11
70.07
141.10
169.10
241.07
113.04
168.10
182.12
240.07
196.10
228.07
329.09
100.08
345.11
144.10
56.05

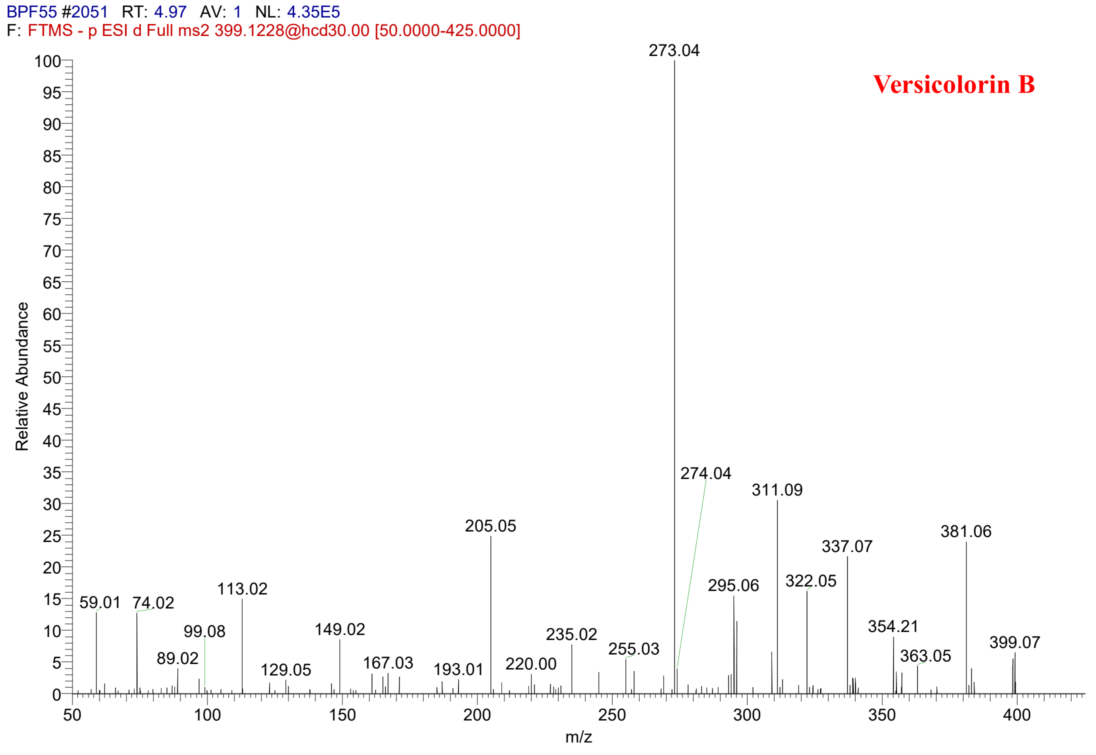
BPF55 #2051 RT: 4.97 AV: 1 NL: 4.35E5
F: FTMS - p ESI d Full ms2 399.1228@hcd30.00 [50.0000-425.0000]
Versicolorin B
Relative Abundance
m/z
273.04
274.04
311.09
205.05
381.06
337.07
295.06
322.05
113.02
59.01
74.02
99.08
149.02
235.02
354.21
399.07
89.02
255.03
167.03
363.05
220.00
193.01
129.05

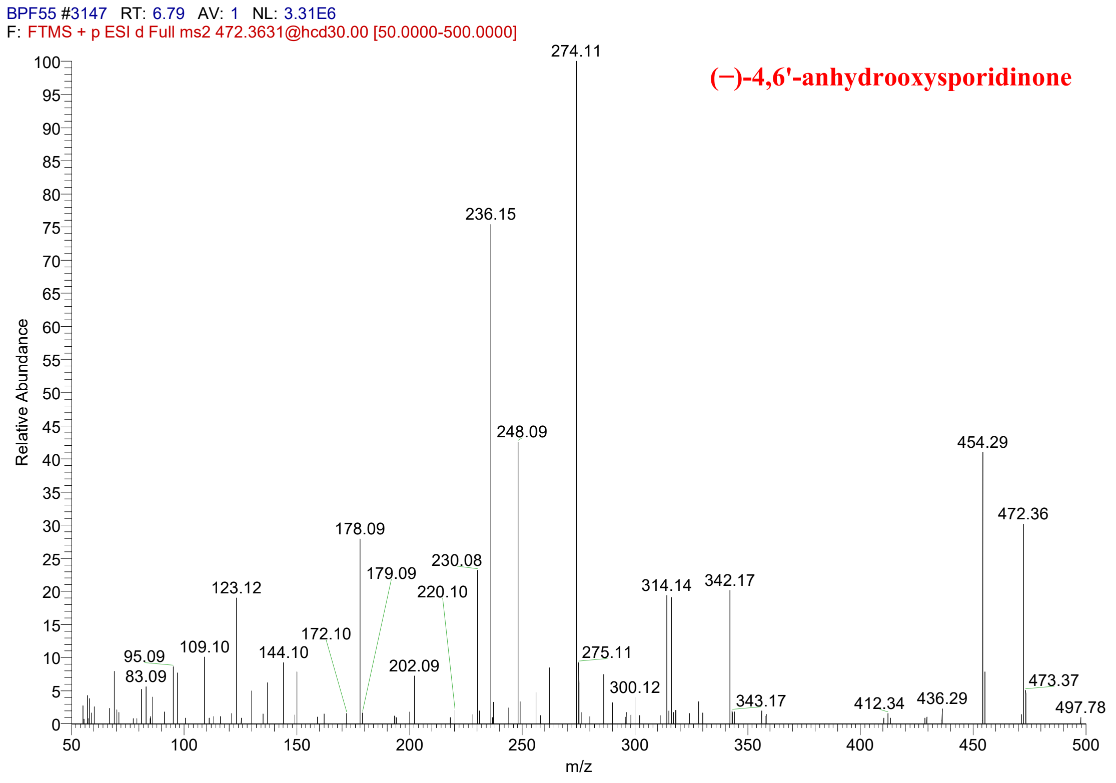
BPF55 #3147 RT: 6.79 AV: 1 NL: 3.31E6
F: FTMS + p ESI d Full ms2 472.3631@hcd30.00 [50.0000-500.0000]
(−)-4,6'-anhydrooxysporidinone
Relative Abundance
m/z
274.11
236.15
248.09
454.29
472.36
178.09
179.09
230.08
220.10
123.12
314.14
342.17
172.10
109.10
144.10
95.09
83.09
202.09
275.11
300.12
343.17
412.34
436.29
473.37
497.78

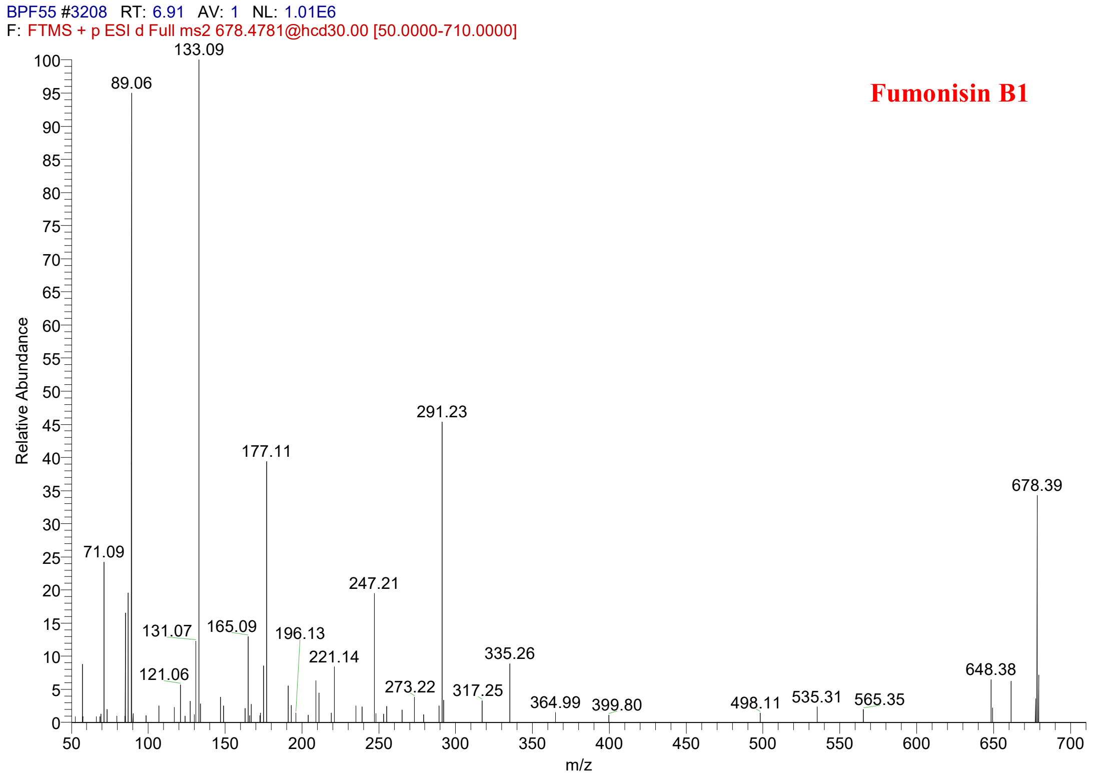
BPF55 #3208 RT: 6.91 AV: 1 NL: 1.01E6
F: FTMS + p ESI d Full ms2 678.4781@hcd30.00 [50.0000-710.0000]
Fumonisin B1
Relative Abundance
m/z
133.09
89.06
291.23
177.11
678.39
71.09
247.21
131.07
165.09
196.13
221.14
121.06
335.26
648.38
273.22
317.25
364.99
399.80
498.11
535.31
565.35

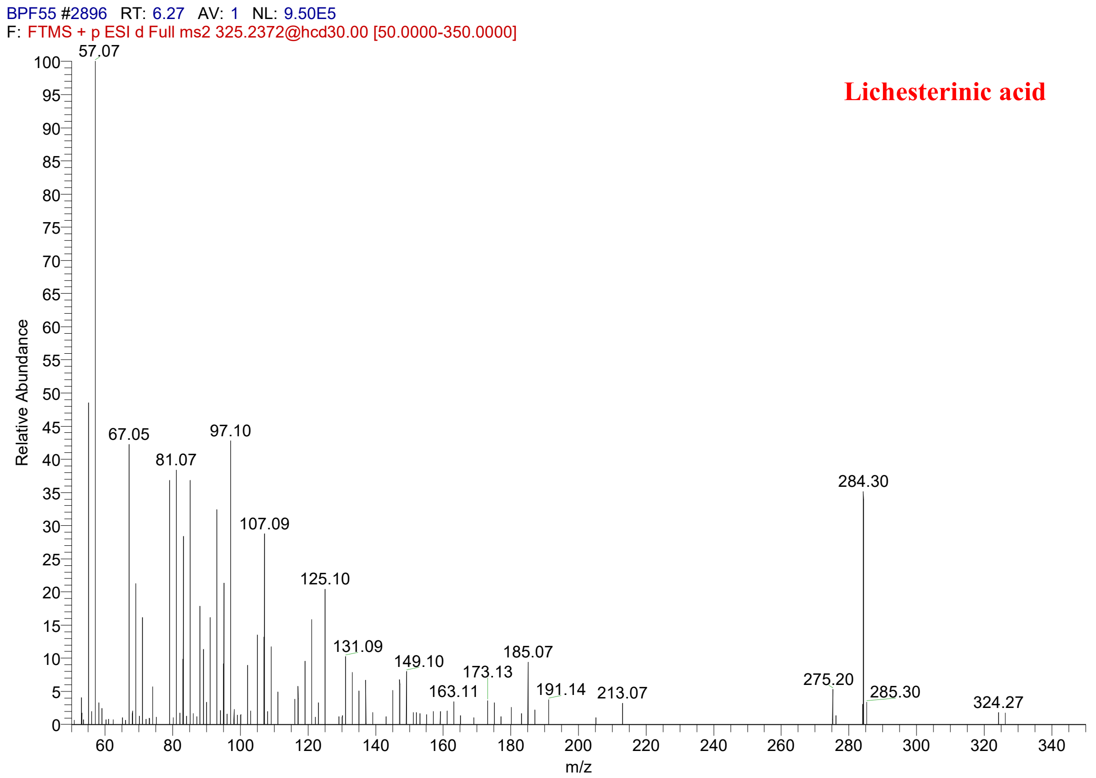

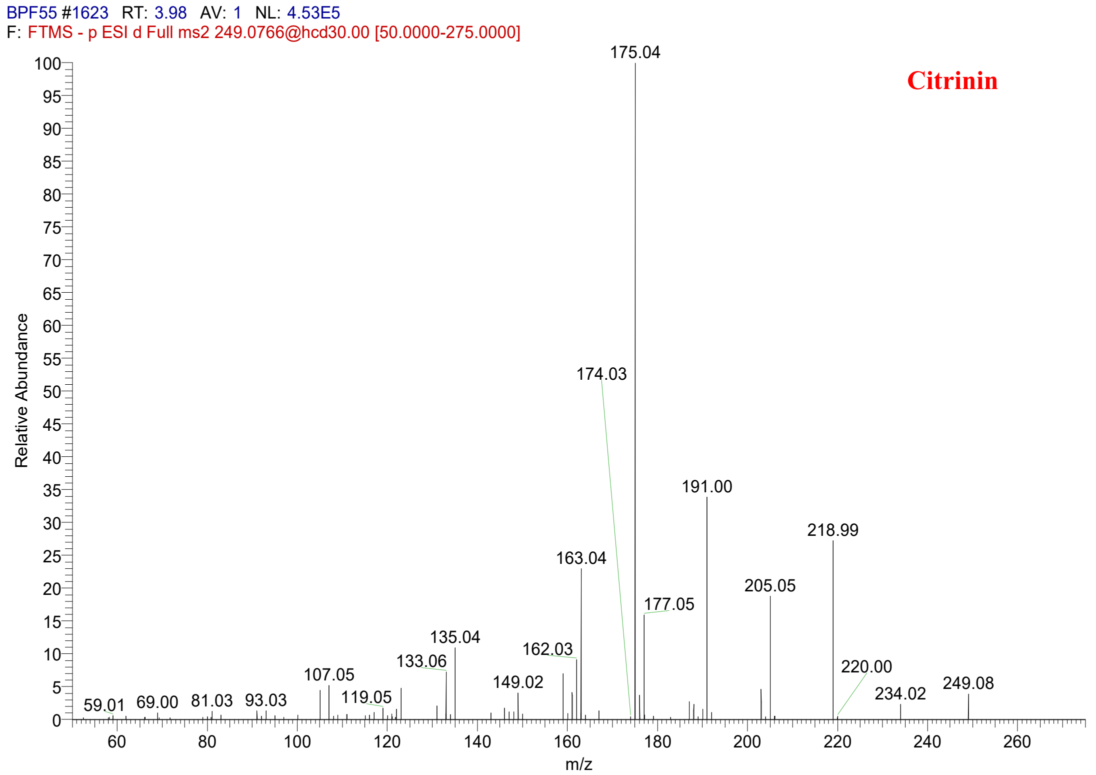

Figure S2. The positive mode spectra peaks of the UPLC-MS/MS analysis for the ten metabolites produced by *F. oxysporum* BPF55

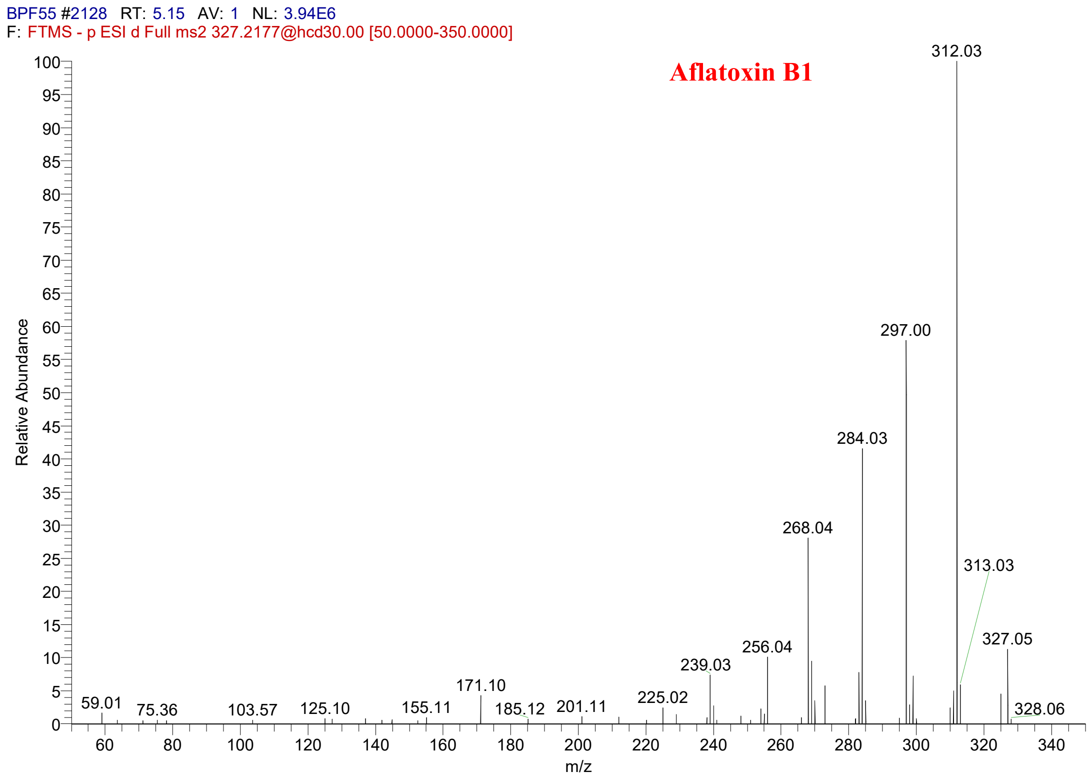
BPF55 #2128 RT: 5.15 AV: 1 NL: 3.94E6
F: FTMS - p ESI d Full ms2 327.2177@hcd30.00 [50.0000-350.0000]
Aflatoxin B1
Relative Abundance
m/z
312.03
297.00
284.03
268.04
313.03
327.05
256.04
239.03
171.10
225.02
59.01
75.36
103.57
125.10
155.11
185.12
201.11
328.06

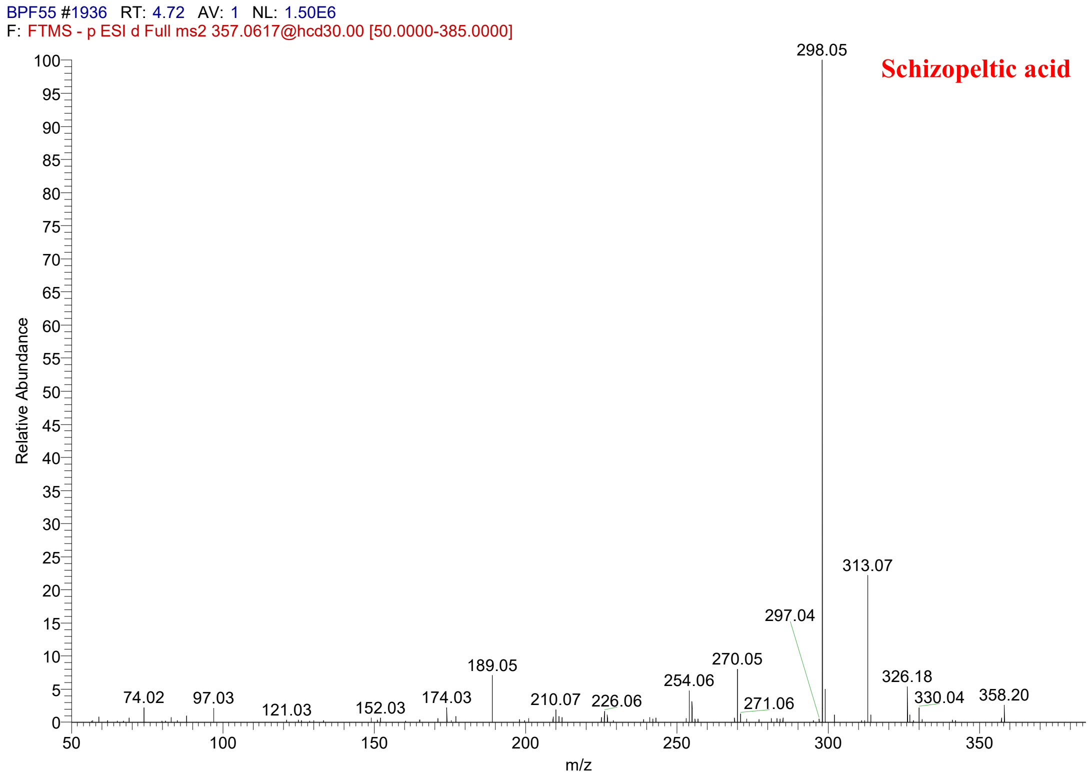
BPF55 #1936 RT: 4.72 AV: 1 NL: 1.50E6
F: FTMS - p ESI d Full ms2 357.0617@hcd30.00 [50.0000-385.0000]
Schizopeltic acid
Relative Abundance
m/z
298.05
313.07
297.04
270.05
189.05
254.06
326.18
74.02
97.03
121.03
152.03
174.03
210.07
226.06
271.06
330.04
358.20

BPF55 #2101 RT: 5.09 AV: 1 NL: 5.20E5
F: FTMS - p ESI d Full ms2 397.0563@hcd30.00 [50.0000-425.0000]

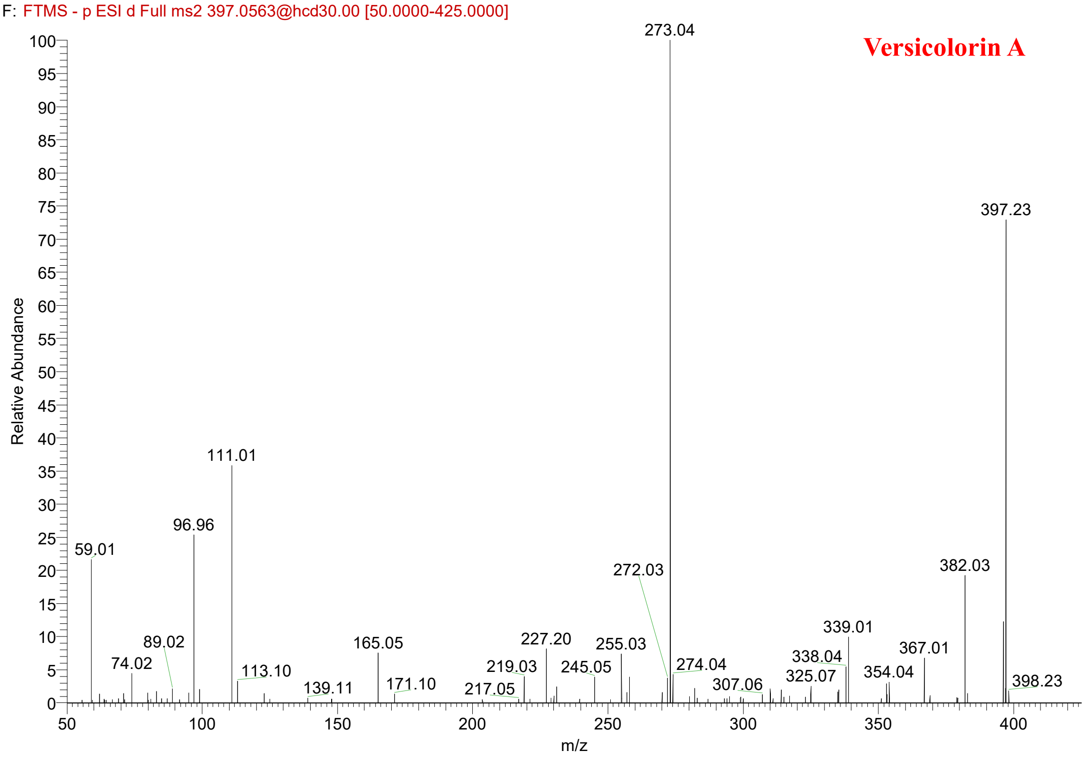

BPF55 #2448 RT: 5.88 AV: 1 NL: 3.57E5
F: FTMS - p ESI d Full ms2 369.1588@hcd30.00 [50.0000-395.0000]

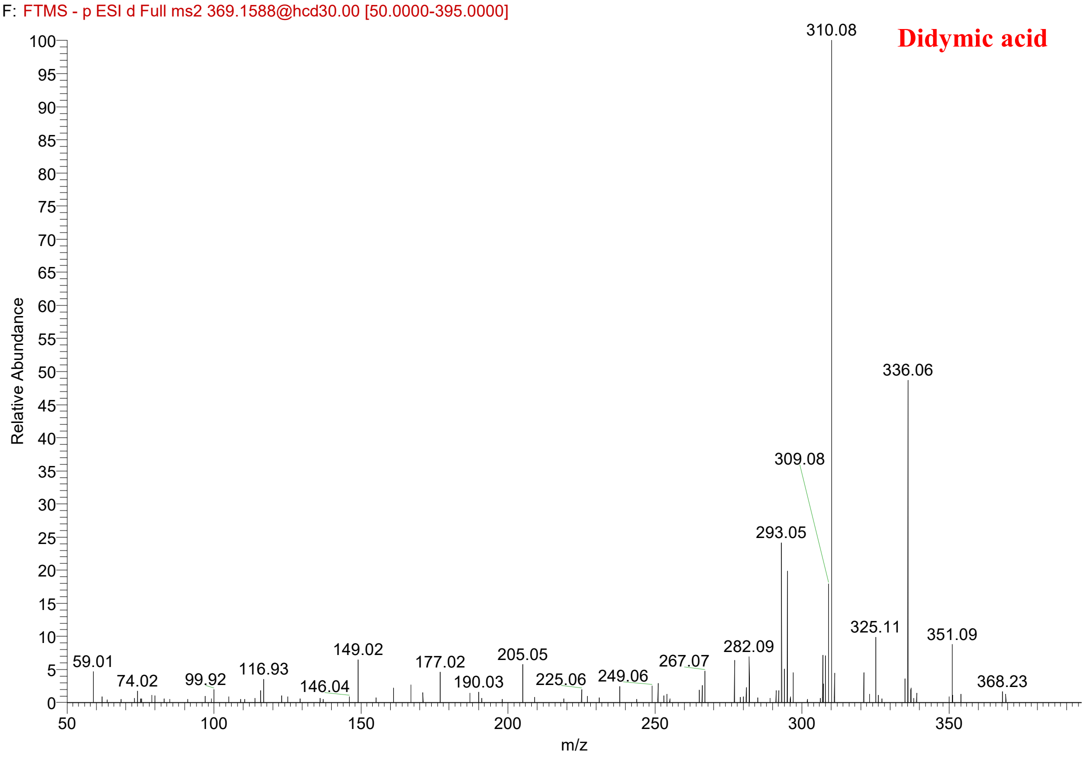

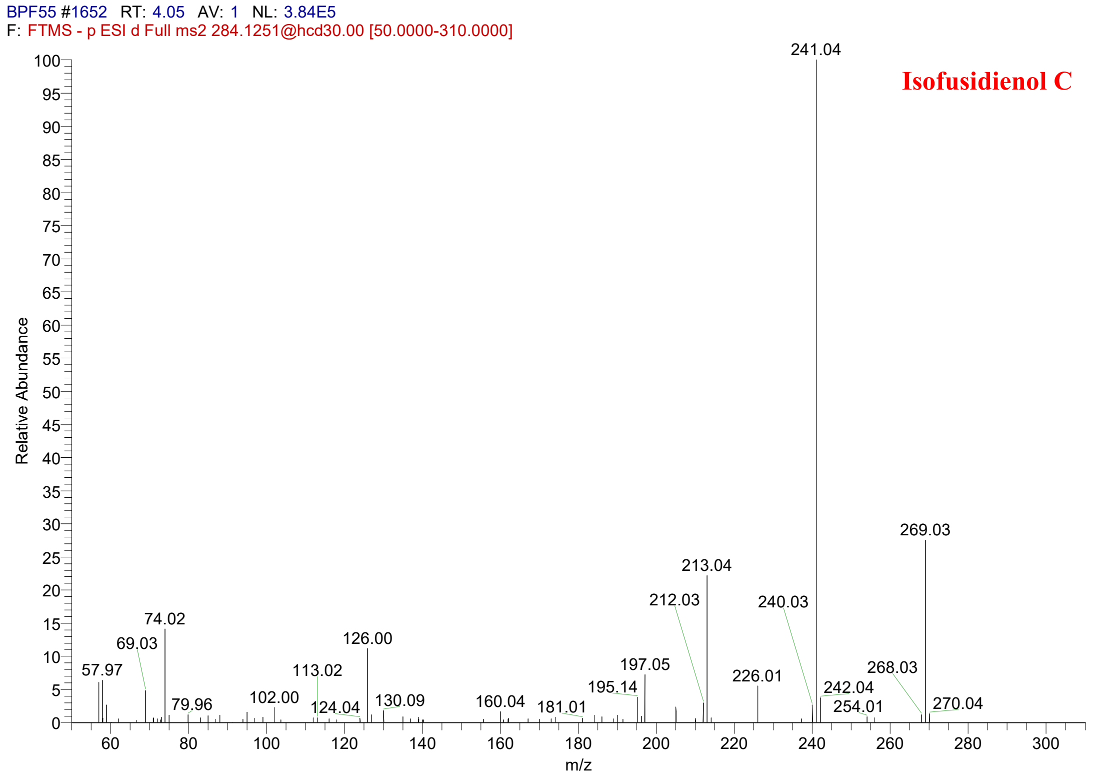

Figure S3. The negative mode spectra peaks of the UPLC-MS/MS analysis for the five metabolites produced by *F. oxysporum* BPF55
